# Limitations in the Design of Chimeric Antigen Receptors for Cancer Therapy

**DOI:** 10.3390/cells8050472

**Published:** 2019-05-17

**Authors:** Stefan Stoiber, Bruno L. Cadilha, Mohamed-Reda Benmebarek, Stefanie Lesch, Stefan Endres, Sebastian Kobold

**Affiliations:** 1 Center of Integrated Protein Science Munich (CIPS-M) and Division of Clinical Pharmacology, Department of Medicine IV, University Hospital, Ludwig-Maximilians-Universität München, Member of the German Center for Lung Research (DZL), 80337 Munich, Germany; stefan.stoiber@campus.lmu.de (S.S.); bcadilha@gmail.com (B.L.C.); reda.benmebarek@gmail.com (M.-R.B.); steffi-lesch@t-online.de (S.L.); Stefan.Endres@med.uni-muenchen.de (S.E.); 2 German Center for Translational Cancer Research (DKTK), 80337 Munich, Germany

**Keywords:** CAR T cell, chimeric antigen receptor, immunotherapy, adoptive cell therapy

## Abstract

Cancer therapy has entered a new era, transitioning from unspecific chemotherapeutic agents to increasingly specific immune-based therapeutic strategies. Among these, chimeric antigen receptor (CAR) T cells have shown unparalleled therapeutic potential in treating refractory hematological malignancies. In contrast, solid tumors pose a much greater challenge to CAR T cell therapy, which has yet to be overcome. As this novel therapeutic modality matures, increasing effort is being invested to determine the optimal structure and properties of CARs to facilitate the transition from empirical testing to the rational design of CAR T cells. In this review, we highlight how individual CAR domains contribute to the success and failure of this promising treatment modality and provide an insight into the most notable advances in the field of CAR T cell engineering.

## 1. Introduction

**Figure 1:**
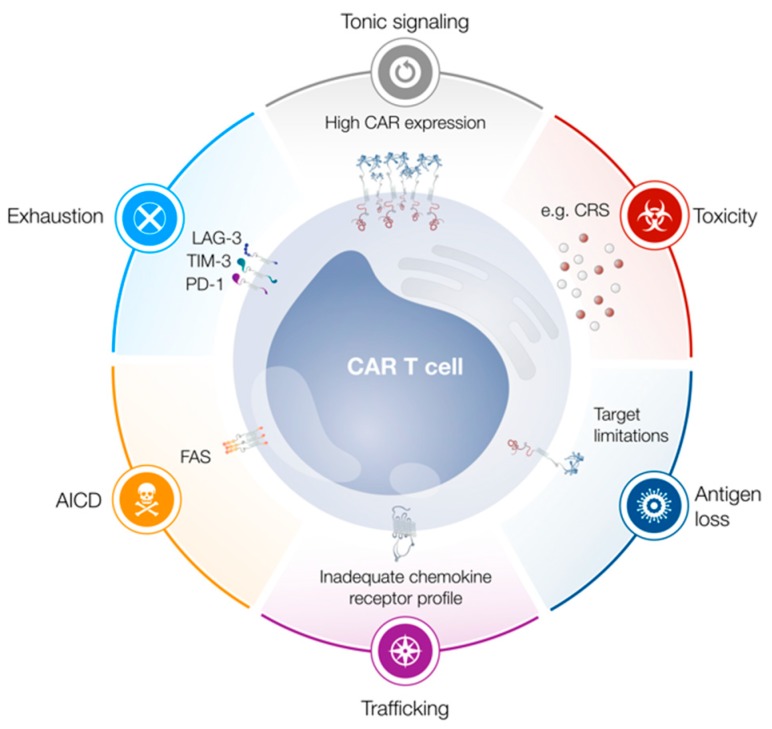
Limitations of chimeric antigen receptor (CAR) T cells. Tonic signaling, exhaustion and activation-induced cell death (AICD) limit T cell functionality, proliferation and persistence. Trafficking of CAR T cells to the tumor site may be limited due to an inadequate chemokine receptor profile. Antigen loss can lead to tumor escape, while cytokine release syndrome (CRS) constitutes a frequently observed adverse event. Abbreviations: PD-1, programmed cell death protein 1; LAG-3, lymphocyte activation gene 3; TIM-3, T cell immunoglobulin mucin 3.

The ability of immune cells to detect and destroy cancer cells forms the basis of all modern immunotherapies, including cancer vaccines, checkpoint blockade, and adoptive cell transfer (ACT). These distinct approaches to immunotherapy have been extensively reviewed elsewhere [1,2,3]. ACT relies on the ability to generate large numbers of tumor-specific T cells. This may be achieved by isolating tumor infiltrating lymphocytes (TILs) or by genetically modifying peripheral blood lymphocytes (PBLs) for cancer specificity. Tumor-reactive T cells can be generated from PBLs either through the introduction of a specific T cell receptor (TCR) or a fully synthetic receptor, usually referred to as chimeric antigen receptor. Thereafter, T cells may be expanded ex vivo and reinfused into the patient, with the ultimate goal to eradicate cancer cells and provide long-lived immunological memory. However, each of these approaches to ACT presents a unique set of obstacles. Successful TIL therapy relies on tumors to elicit an endogenous immune response, and this approach may therefore be less suitable for immunologically “cold” tumors with low numbers of infiltrating immune cells [4,5]. Furthermore, T cells isolated from the tumor microenvironment are often terminally differentiated and functionally exhausted [5], while TIL therapy may altogether not be applicable to patients with inaccessible or unresectable tumors. Genetic engineering may overcome some of these challenges, as the specificity of T cells isolated from peripheral blood can be modulated as necessary. If these polyclonal cells are modified to express a tumor-specific TCR, major histocompatibility complex (MHC)-restricted antigen recognition allows for tumor escape via disruption of antigen processing or presentation [6,7]. In contrast, CAR T cells couple the specificity of an antibody with the destructive force of T cell effector functions [8], thereby constituting a powerful approach to ACT. CAR T cells were first described in the late 1980s [9,10] and have since garnered much attention. A CAR usually consists of an antibody-derived single-chain variable fragment (scFv), which is linked via a spacer and transmembrane domain to intracellular signaling molecules, capable of eliciting T cell effector functions. Originally, first-generation CAR T cells contained only a CD3ζ intracellular domain, capable of recapitulating “signal 1” of T cell activation. However, first-generation CAR T cells displayed poor anti-tumor efficacy in patients, owing to the limited expansion and persistence of transferred T cells [11,12,13]. The inclusion of one or multiple costimulatory domains gave rise to second- or third-generation CARs, respectively, and is intended to enhance T cell function upon antigen recognition. Several clinical studies have reported dramatic response rates in relapsed or refractory (r/r) hematological malignancies, showcasing the unparalleled therapeutic potential of anti-CD19-CAR T cells to treat diseases such as acute lymphoblastic leukemia (ALL) [14], diffuse large B cell lymphoma (DLBCL) [15] and, to a lesser extent, chronic lymphocytic leukemia (CLL) [16]. The clinical success of CAR T cell therapy eventually culminated in the FDA approval of two CD19-specific CAR T cell products, namely tisagenlecleucel for r/r ALL and r/r large B cell lymphoma and axicabtagene cliloleucel for r/r large B cell lymphoma [17,18]. However, various CAR T cell-mediated toxicities, such as tumor lysis syndrome [19,20], cytokine release syndrome [19,21,22], neurotoxicity [23,24] and on-target off-tumor toxicity [25,26,27,28] have emerged, some with devastating consequences. Furthermore, antigen loss and the consequent tumor escape limit the long-term success of CAR T cell therapy in a significant fraction of patients [29]. So far, CAR T cells have lacked potent clinical efficacy when targeting solid tumors. This is likely due to numerous hindrances, most notably CAR T cell dysfunction in a hostile tumor microenvironment, limited trafficking of CAR T cells to the tumor site and antigen heterogeneity amongst tumor cells [30,31,32]. Additionally, antigen-independent tonic CAR signaling has been frequently demonstrated to harbor deleterious consequences for CAR T cells, potentially contributing to the therapeutic failure of a number of clinical CAR candidates [33,34,35,36,37]. However, in some cases, tonic signaling may confer an antigen-independent proliferative advantage to modified T cells as well as improved in vivo persistence or efficacy [38,39]. Accurately defining tonic CAR signaling has proven difficult, with a multitude of different receptors inducing distinct phenotypic or functional changes in CAR T cells. At the same time, multiple CAR domains have been reported to contribute to this complex phenomenon. Figure 1 gives an overview of the most notable limitations of CAR T cells.

**Figure 2:**
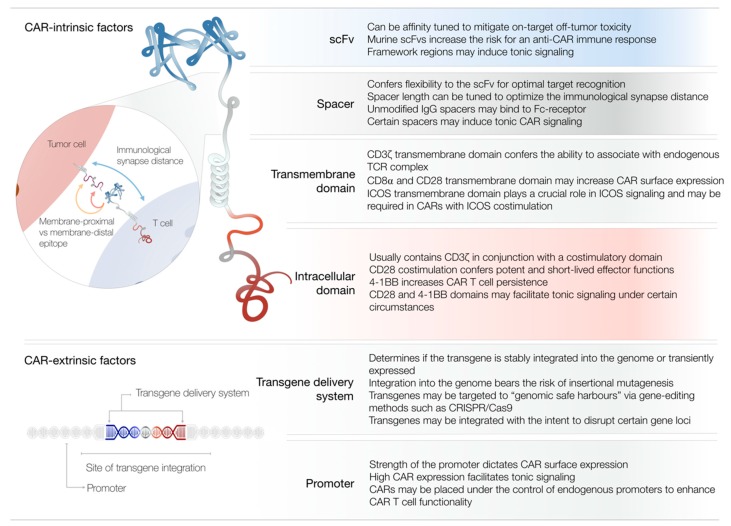
Summary of CAR-intrinsic and CAR-extrinsic factors of CAR T cell design. Depicted is a representation of a CAR molecule with its respective domains. The table lists the most notable contributions of individual domains to CAR T cell functionality. The zoom-in illustrates how the length of the spacer domain may influence the immunological synapse distance and confer flexibility for optimal target recognition. Abbreviations: Ig, immunoglobulin; ICOS, inducible T cell costimulator; CRISPR, clustered regularly interspaced short palindromic repeats.

In the following sections, we will provide a comprehensive discussion of individual CAR domains and their respective contributions to CAR T cell functionality (see Figure 2 for a summary). We will highlight how advances in gene editing may revolutionize CAR T cell engineering and provide an outlook on novel CAR formats designed to overcome various obstacles on the way to successful CAR T cell therapy.

## 2. Antigen Recognition Domain

The antigen-binding properties of a CAR are defined by the antigen recognition domain, which usually consists of a single-chain variable fragment (scFv). The feasibility of generating a scFv through linking the variable light (V_L_) and variable heavy (V_H_) regions of a monoclonal antibody by a short linker was first demonstrated in 1988 and scFvs normally retain the specificity and affinity of the original antibody [40,41]. Conceptually, one might assume that the configuration in which V_L_ is followed by the linker then by V_H_ more closely mimics the natural antibody design and would therefore be superior. However, empirical testing has revealed that both V_L_–linker–V_H_ and V_H_–linker–V_L_ configurations can function properly. In some cases, one particular orientation may confer improved expression and functional superiority over the other [42,43]. Similarly, different linker molecules have been successfully utilized in designing scFvs. Currently, the majority of linkers used in CAR T cells encompass some variation of a polypeptide based on glycine (Gly) and serine (Ser) repeats. For example, the (Gly_4_Ser)_3_–linker consists of three repeats of the pentapeptide Gly–Gly–Gly–Gly–Ser. The use of these residues is intended to confer flexibility and minimize the risk for interference of the linker with the proper folding and function of the connected protein domains [41,44,45]. Some necessary requirements for linker length may be inferred from studies of free scFv molecules, as linkers which are too short favor the formation of scFv multimers [46,47]. In the context of scFv-based CAR T cells, this might facilitate tonic signaling through antigen-independent CAR clustering [33]. It is generally thought that the optimal linker length is within the range of 15–20 amino acids, and many recent CAR constructs have indeed utilized (Gly_4_Ser)_3_ or (Gly_4_Ser)_4_ linkers, respectively [43,48,49,50].

A crucial determinant of CAR functionality is the affinity of the scFv for its cognate antigen. Generally, scFv-based CAR T cells have an affinity for their target which is, on average, several orders of magnitude greater than that of unmodified TCR T cells. However, molecules such as CD4, CD8 and the CD3 chains γ, δ and ε are absent in CARs, but contribute to the activation signal delivered through the TCR [51]. The optimal antigen-affinity of CAR T cells likely varies based on many factors, such as costimulatory domains and spacer design, and has indeed been shown to depend on the antigen density on the target cell, as well as CAR expression by the T cell [52]. Work done by several groups suggests that there is a lower limit to CAR affinity, below which sufficient antigen recognition does not occur, resulting in suboptimal CAR T cell activation [53,54]. However, especially against high antigen-expressing target cells, increasing CAR affinity does not necessarily improve CAR T cell function, implying the existence of an upper limit to CAR affinity which may be unique to any given level of antigen expression [52,55].

The fine-tuning of CAR affinity also encompasses the possibility to mitigate on-target off-tumor toxicities related to low-level antigen expression on normal tissue, while retaining sufficient effector functions to eradicate antigen-overexpressing malignant cells [52,55,56,57]. A cornerstone of this rationale was provided by Liu and colleagues, who generated several CAR variants with different affinities for human epidermal growth factor receptor 2 (HER2/neu). Low-affinity CAR T cells demonstrated robust anti-tumor efficacy both in vitro and in a xenograft mouse model, meanwhile sparing primary cell lines expressing physiological levels of HER2/neu [52]. This attenuated response to normal cells is especially relevant in light of a previous report, where a patient died after CAR T cell therapy, likely due to recognition of lung epithelial cells expressing low levels of HER2/neu [26].

Another distinct advantage of scFv-based CAR T cells is the ability to recognize their antigen independently of MHC presentation. This prevents tumor escape via the downregulation of MHC molecules [6,7] and confers CAR T cells with the ability to recognize non-peptide antigens such as glycolipids or tumor-specific glycosylation patterns [58,59]. The initial drawback of this MHC-independent recognition was the restricted targeting of surface antigens. However, since it is possible to generate antibodies against specific peptide–MHC complexes, so called TCR-mimic CAR T cells have been conceived and extend the possible range of CAR targets to the vast array of intracellular proteins [48,60]. However, loss or downregulation of the target antigen will inevitably result in treatment failure, as CAR T cells become agnostic to the cancer cells [29]. This issue is currently being addressed, with the emergence of CAR T cells targeting more than one antigen on tumor cells [61,62].

ScFv-based CAR T cells can theoretically be redirected towards any antigen, provided the amino acid sequence of an antibody with the desired specificity is known. However, Long and colleagues have shown that there may be distinct properties intrinsic to some scFvs that limit their application in CAR T cells [33]. By comparing two CARs directed against either CD19 (FMC63-scFv) or GD2 (14g2a-scFv), they found that the framework regions of the 14g2a-scFv induced antigen-independent clustering of anti-GD2-CARs on the T cell surface, resulting in tonic CAR signaling. This promoted the rapid exhaustion of anti-GD2-CAR T cells during in vitro culture, consequently limiting their ability to properly function in vivo. However, attempts by the authors to rescue the anti-GD2 CAR by replacing the 14g2a framework regions for the ones found in the FMC63-scFv resulted in failed CAR expression, demonstrating that framework regions may not always be exchangeable.

One issue, which remains mostly unresolved, is that the majority of clinical CAR trials have utilized scFvs derived from murine antibodies, thereby increasing the risk for an anti-CAR T cell immune response, which may cause toxicity or limit CAR T cell persistence [63,64]. This problem may be addressed by humanizing murine scFvs [65,66] or by deriving scFvs from fully human antibodies [57,67]. This allows for the generation of conceivably less immunogenic CAR T cells, which have been shown to induce remission of ALL refractory to previous murine-scFv-based anti-CD19-CAR T cell therapy [66]. However, owing to the chimeric nature of these receptors, even constructs which are derived exclusively from human proteins may elicit a host immune response. This response would result from the creation of immunogenic peptide sequences at junction sites of CAR domains or from the induction of anti-idiotype antibodies [49].

Besides scFvs, other targeting moieties have been tested in CAR T cells [68,69,70,71], but an in-depth discussion of these alternative antigen-recognition domains is beyond the scope of this review and can be found elsewhere [72,73].

## 3. Spacer Domain

The spacer (also referred to as hinge) connects the scFv to the transmembrane domain and most CAR constructs have been devised with either immunoglobulin G (IgG)-based hinges or derivatives of CD8α or CD28 extracellular domains. The optimal spacer length has been subject to intensive investigation and appears to depend both on the antigen and the position and accessibility of the targeted epitope. Several groups have shown that CAR T cells are more potently activated if the target epitope resides closer to the target cell membrane [74,75,76]. This observation has led to the hypothesis that tuning the length of the spacer may place the CAR T cell and target cell at the optimal intercellular distance to exclude large phosphatases such as CD45 during immunological synapse formation, which may otherwise attenuate CAR signaling [74,77,78]. Indeed, several groups have observed that short spacer CARs targeting CD19, carcinoembryonic antigen (CEA), interleukin-13 receptor alpha-2 (IL13Rα2) and membrane distal epitopes within the receptor tyrosine kinase-like orphan receptor 1 (ROR1) induce stronger T cell activation than CARs with long spacer domains [53,79,80,81]. However, in some cases, the targeted epitope may be relatively inaccessible, necessitating the use of longer spacers, conceivably to overcome steric hindrances and to enable effective antigen binding through the scFv. In fact, various groups have demonstrated the need for longer spacers in CARs targeting inaccessible or membrane-proximal epitopes within ROR1, mucin 1 (MUC1), neural cell adhesion molecule (NCAM) and the oncofetal antigen 5T4 [79,80,82]. Therefore, the optimal spacer length varies depending on the targeted epitope and may have to be adjusted accordingly when targeting novel antigens.

One distinct problem arising from human IgG-derived spacers is that, if unedited, they retain their ability to bind to Fc-receptor (FcγR)-bearing cells via their heavy chain constant 2 (C_H_2) domain, leading to the off-target activation of CAR T cells [37,80,83,84]. Likewise, this unwanted interaction between immune cells has been shown to activate human monocytic cells as well as natural killer (NK) cells in vitro [83]. Several deleterious consequences of utilizing unedited IgG-derived spacers, such as sequestration of CAR T cells in the lung [37,80], activation-induced cell death (AICD) resulting in limited T cell persistence [80], and toxicity mediated by off-target CAR T cell activation [84], have been observed in mouse models. These effects were shown to be similar for IgG1- as well as IgG4-based hinges [37,80,83,85]. However, many groups were able to abrogate spacer-binding to FcγR by deleting the C_H_2 domain or by mutating certain amino acids that are essential to FcγR-binding [37,80,83,85]. Therefore, the negative consequences of incorporating IgG-based hinges may be avoided, and the modular composition of IgG-derived hinges can be used to generate a number of differently sized spacers, commonly referred to as long, intermediate and short spacers, to optimize CAR structure [37,50,53].

It is unclear to which degree these preclinical findings would translate to patients, since the above-mentioned studies were exclusively carried out in mice and the encountered problems relied upon binding of spacers to the murine FcγR. Several clinical studies utilizing CAR T cells with unedited IgG-derived hinges have been conducted without conclusive evidence for FcγR-related issues [11,13,86,87]. However, all of these studies reported limited CAR T cell persistence and overall modest anti-tumor efficacy and in one case the authors speculate that this may in part be due to the unedited IgG-hinge [87]. Similarly, Brown and colleagues reported low persistence and limited anti-tumor activity of anti-IL13Rα2-CAR T cells with an unmodified IgG4-hinge in patients with glioblastoma [68]. Intriguingly, the same group later reported regression of recurrent multifocal glioblastoma in one patient following anti-IL13Rα2-CAR T cell therapy [88]. This time, the authors used a mutated IgG4-based hinge to reduce off-target Fc-receptor interactions, providing another clue as to the importance of spacer design. Nevertheless, while this patient had a remarkable clinical response, the expansion and persistence of CAR T cells still appeared to be limited. Furthermore, multiple variables besides the choice of hinge region differed between these studies (e.g., the inclusion of a 4-1BB costimulatory domain and the CAR T cell manufacturing process), prohibiting a conclusive verdict about the impact of IgG-derived hinges on clinical CAR T cell efficacy.

Although CD8α and CD28 spacers have been extensively utilized in numerous clinical anti-CD19-CAR trials demonstrating potent anti-tumor efficacy [14,19,20,89], the impact of these hinges on CAR T cell performance has not been thoroughly investigated as of yet, thus their use remains largely empirical. Some limited evidence regarding the utility of these domains was provided by Alabanza and colleagues [67]. The authors compared different second-generation anti-CD19-CARs incorporating either a CD8α-derived hinge and transmembrane region (CD8-28ζ-CAR) or a CD28-based hinge and transmembrane region (28ζ-CAR). While CD8-28ζ-CAR T cells produced lower levels of cytokines in response to CD19^+^ cells in vitro, they also experienced lower levels of AICD compared to 28ζ-CAR T cells after repeated exposure to antigen. Importantly, CD8-28ζ-CAR T cells demonstrated equal efficacy as 28ζ-CAR T cells in eliminating tumors from mice with the potential for less cytokine-mediated toxicity. Following a comparison of the crystal structures of the extracellular domains of CD8α and CD28 the authors hypothesized that the observed differences in CAR signal strength stem from the greater propensity of CD28-based hinges to facilitate CAR dimerization. However, due to the fact that the hinge regions were exchanged simultaneously with the transmembrane domain, it remains difficult to discern the relative contribution of each domain to the functional differences observed in this study. This distinction may be especially relevant in light of the data provided by Morin and colleagues, which demonstrated that truncated CD28 molecules, lacking an intracellular domain, may still contribute to T cell activation [90].

Finally, it has been implied that certain spacers promote tonic CAR signaling. Frigault and colleagues demonstrated that different CARs targeting mesothelin or the tyrosin-protein kinase Met (c-Met) induce tonic signaling in T cells. The authors mainly attributed this phenomenon to high CAR surface expression in conjunction with certain scFvs and the CD28 costimulatory domain. However, it is noteworthy that the exchange of the IgG4-hinge in a c-Met-specific CAR for a CD8α spacer appeared to completely abrogate tonic signaling, while mesothelin-specific CAR T cells displayed tonic signaling independently of the respective spacer domain [36]. Similarly, Watanabe and colleagues observed that the inclusion of a modified IgG1-C_H_2C_H_3 spacer domain in a prostate stem cell antigen (PSCA)-specific CAR promoted antigen-independent T cell activation and secretion of cytokines as well as accelerated T cell differentiation in vitro. Consequently, CAR T cells demonstrated modest anti-tumor efficacy in vivo. When the authors replaced the long C_H_2C_H_3 spacer for an intermediate C_H_3 spacer, tonic signaling was abrogated and the anti-tumor efficacy of CAR T cells was markedly enhanced [37].

In light of the data provided by Long and colleagues, it is tempting to hypothesize that a longer hinge may potentiate the intrinsic ability of some scFvs to oligomerize, thus promoting tonic CAR signaling [33].

In conclusion, these studies highlight the complex nature of the spacer domain and encourage further investigation to determine the optimal spacer structure for improved CAR design.

## 4. Transmembrane Domain

The transmembrane domain links the extracellular domains of the CAR to the intracellular signaling domains and anchors the receptor to the T cell membrane. Commonly used transmembrane domains have been derived from CD4, CD8α, CD28 and CD3ζ [67,86,88,91]. Due to the fact that in many studies transmembrane domains are exchanged concomitantly with other domains (e.g., spacers or costimulatory domains), it is profoundly difficult to make an accurate statement regarding their contribution to CAR functionality. There is however some limited data that points towards distinct advantages of utilizing certain transmembrane domains to enhance CAR surface expression or CAR T cell performance.

Bridgeman and colleagues demonstrated early on that the CD3ζ transmembrane domain endowed a first-generation CAR with the ability to form homodimers as well as heterodimers with the endogenous TCR-complex [92]. This interaction led to increased responsiveness of CAR T cells to CEA, while mutagenesis of key amino acids within the transmembrane domain diminished CAR T cell function. The use of the CD3ζ transmembrane domain has become uncommon in second and third-generation CARs, which deliver a potent stimulus to the T cell without the need to associate with the endogenous TCR-complex. Furthermore, a subsequent report [91] suggested that the CD8α as well as the CD28 transmembrane domain increased the surface expression of a first-generation CAR compared to the CD3ζ domain, potentially explaining the widespread use of CD8α and CD28 transmembrane domains in many successful clinical trials [14,19,64,89].

Intriguingly, Guedan and colleagues recently showed that a third-generation anti-mesothelin-CAR with inducible T cell costimulator (ICOS) and 4-1BB costimulatory domains required the ICOS transmembrane domain to eradicate tumors in a xenograft mouse model [35]. When the same CAR was constructed with the CD8α transmembrane domain, the anti-tumor efficacy was substantially reduced, owing to the limited expansion and persistence of CAR T cells. This marked contribution of the transmembrane domain to CAR functionality may be unique to ICOS, as a recent report from Wan and colleagues demonstrated the important role of the ICOS transmembrane domain for ICOS signaling [93]. These results warrant further investigation and highlight the crucial impact this supposedly inert domain could have on CAR T cell performance.

Considering the limited availability of data, it seems reasonable to suggest the use of transmembrane domains in conjunction with their respective intracellular costimulatory or spacer domains until the functional contribution of this understudied domain has been thoroughly investigated, and a more rational choice can be made.

## 5. Intracellular Costimulatory Domain

Costimulatory domains are usually derived from either the CD28 receptor family (CD28, ICOS) or the tumor necrosis factor receptor family (4-1BB, OX40, CD27). An in-depth analysis of the signaling cascades initiated by these costimulatory domains is beyond the scope of this review and has been discussed elsewhere [94]. Herein, we will review the most notable contributions of individual costimulatory domains to CAR T cell performance and highlight how certain CAR designs may preferentially confer distinct functional properties to CAR T cells.

### 5.1. Second-Generation CAR T Cells

While the inclusion of a costimulatory domain does little to improve the in vitro cytolytic function of second-generation CAR T cells, the secretion of cytokines, CAR T cell proliferation and overall anti-tumor efficacy are markedly enhanced compared to first-generation CAR T cells [38,95,96,97,98]. The superior expansion and persistence of second-generation CAR T cells compared to first-generation CAR T cells has furthermore been demonstrated in patients [13].

The most widely used costimulatory domains are derived from CD28 and 4-1BB, both of which have been extensively characterized in head-to-head comparisons. Preclinically, CD28 costimulation was shown to promote the rapid development of T cell effector functions [97,98] but conferred limited in vivo T cell persistence compared to CARs incorporating 4-1BB [38,98]. Conversely, 4-1BB-containing CAR T cells displayed slower tumor eradication kinetics in a mouse model of leukemia, but accumulated over time and ultimately demonstrated comparable anti-tumor efficacy [98]. Some mechanistic insight into the divergent response kinetics and persistence of CD28- and 4-1BB-containing CAR T cells has since been provided by Kawalekar and colleagues. The authors compared both CD19- and mesothelin-specific second-generation CAR T cells utilizing either CD28 or 4-1BB costimulation (CD28-CAR, 4-1BB-CAR) and found that the use of either costimulatory domain distinctly reprogrammed T cell metabolism upon CAR activation [99]. CD28 costimulation skewed CAR T cell differentiation towards an effector-memory type, with CD28-CAR T cells predominantly relying on glycolytic metabolism. Contrarily, 4-1BB costimulation promoted mitochondrial biogenesis, enhanced respiratory capacity and increased fatty acid oxidation. Moreover, upon antigenic stimulation, 4-1BB-CAR T cells preferentially differentiated into central memory T cells. This data indicates that the rapid and potent effector functions of CD28-containing CAR T cells may in part reflect their increased utilization of glucose to cover their metabolic demands (a hallmark of effector T cells), while 4-1BB-containing CAR T cells may have superior persistence due to their increased capacity for oxidative metabolism.

Salter and colleagues have used mass spectrometry to identify phosphorylation events following CAR activation of both CD28- and 4-1BB-containing second-generation CAR T cells [100]. Intriguingly, the authors found that while both CARs induced the phosphorylation of similar signaling intermediates, stronger and faster changes in protein phosphorylation were observed in CD28-CAR T cells. This could in part be explained by the constitutive association of lymphocyte-specific protein tyrosine kinase (Lck) with the CD28-CAR, which was almost absent in the 4-1BB-CAR. The stronger signaling of the CD28-CAR resulted in profound transcriptional changes and the enhanced expression of effector molecules such as granzyme B, interferon-γ (IFN-γ), tumor necrosis factor α (TNF-α) and interleukin 2 (IL-2) upon CAR activation. However, this was accompanied by the acquisition of an exhausted phenotype and reduced anti-tumor efficacy in vivo when lower numbers of CD28-CAR T cells were transferred.

Expanding on these preclinical observations, the clinical trials of second-generation CD28- or 4-1BB-containing CAR T cells have similarly reported disparate results regarding the in vivo persistence of transferred cells. While CD28-based anti-CD19 CAR T cells have shown potent anti-tumor activity, they generally persist for only a limited time [21,24,101] and have therefore been suggested to constitute a potent bridge-to-transplant approach, whereby remission is induced via CAR T cell therapy followed by allogeneic hematopoietic stem cell transplantation (HSCT) [101,102]. In contrast, 4-1BB-containing second-generation CAR T cells have shown similarly potent anti-tumor efficacy, and have been reported to persist for up to several years in patients, potentially conferring long-term immunological memory [29,103]. Likewise, the faster kinetics of effector function reported in preclinical studies may be mirrored by the observation that CAR T cell persistence did not correlate with the survival of patients in a trial utilizing CD28-containing second-generation CAR T cells to treat ALL [24]. In contrast, the clinical trials of second-generation 4-1BB-CAR T cells reported that ALL disease relapse was associated with either a lack of CAR T cell persistence or CD19-negative escape variants [14,64]. However, it is important to bear in mind that the interpretation of cross-study comparisons is error-prone due to potential confounding variables.

Unlike CD28 and 4-1BB, other costimulatory domains have predominantly been characterized preclinically. For example, CD27-based second-generation CAR T cells displayed similar anti-tumor efficacy in a xenograft mouse model of ovarian cancer in direct comparison to CD28- or 41BB-containing CAR T cells. Like 4-1BB, CD27 costimulation increased CAR T cell persistence compared to CD28 [104]. Guedan and colleagues have reported that incorporation of the ICOS costimulatory domain sustains a T_H1_–T_H17_ polarization in CAR T cells and that ICOS-containing CAR T cells showed superior in vivo persistence compared to both 4-1BB- and CD28-based CAR T cells [105]. It remains to be determined whether these alternative costimulatory molecules will provide a clinical benefit over the well-established CD28 and 4-1BB domains.

Besides the incorporation of costimulatory domains into the CAR structure, the selective modification of virus-specific T cells to express a tumor-specific CAR represents an elegant approach to provide additional costimulatory signals for these CAR T cells. In theory, such dual-specific T cells would encounter viral antigens in vivo and therefore would receive additional stimulatory signals after engagement of their endogenous TCR, while retaining anti-tumor specificity via their CAR [106,107]. Indeed, Pule and colleagues showed that Epstein–Barr virus (EBV)-specific T cells engineered to express a first-generation anti-GD2-CAR demonstrated increased T cell persistence in neuroblastoma patients compared to GD2-CAR-T cells without defined TCR specificity [107]. Therefore, the use of virus-specific T cells may present a potential avenue to enhance CAR T cell effectiveness. However, a more recent study demonstrated that the simultaneous in vivo exposure of second-generation CAR T cells to both their CAR antigen and their TCR antigen may lead to exhaustion, apoptosis and diminished anti-tumor function of CD8^+^ CAR T cells [108]. In contrast, CD4^+^ CAR T cells were more resistant to this phenomenon, and retained their anti-tumor capacity for a prolonged period of time [108]. Therefore, further research is needed to elucidate the mechanisms that govern the success of CAR T cells with defined TCR specificity.

### 5.2. Third-Generation CAR T Cells

Third-generation CARs combine two costimulatory domains in one receptor in an attempt to harness the benefits from multiple signaling pathways. Indeed, some preclinical studies have shown the superior function of third-generation CAR T cells in vitro and in vivo compared to second-generation CAR T cells [35,109], while others observed no difference [110] and, in some cases, third-generation CAR T cells performed worse than their second-generation counterparts [50,76,111]. The reasons for this discrepancy are still unclear, and may reflect the fact that certain in vitro surrogate measurements of CAR T cell potency do not always accurately predict T cell functionality in vivo [33,50]. Furthermore, these differential outcomes may in part depend on the need to fine-tune CAR signal strength to mitigate AICD [50].

The observation that extensive CAR signaling can be detrimental to T cell functionality is in alignment with reports of tonic CAR signaling hampering CAR T cell performance. Tonic signaling has been shown to increase with elevated CAR surface expression [34,35,36,112]. Especially, CD28 costimulation has repeatedly been implicated to facilitate tonic CAR signaling [33,36], but tonic 4-1BB signaling has also been observed in certain contexts [34,35].

Interestingly, both beneficial and detrimental tonic CAR signaling has predominantly been reported in second-generation CAR T cells [33,34,36,37,38,39,112,113]. This might in part be explained by a study from Ramello and colleagues, who demonstrated that in contrast to third-generation CAR T cells, second-generation CAR T cells expressed a constitutively phosphorylated form of CD3ζ, irrespective of the costimulatory domain, but dependent on the absolute size of the intracellular domain [114].

The inclusion of two costimulatory domains within one receptor adds further complexity to CAR design, as CAR functionality may differ, depending on the proximity of the respective domains to the cell membrane [35,113]. In this regard, it has recently been shown that 4-1BB-ICOS-ζ-CAR T cells displayed tonic signaling and diminished anti-tumor efficacy in vivo compared to ICOS-4-1BB-ζ-CAR T cells [35]. While tonic signaling appeared to be dependent on the proximity of 4-1BB to the cell membrane, the enhanced anti-tumor efficacy of ICOS-4-1BB-ζ-CAR T cells may have relied solely on the incorporation of the ICOS transmembrane domain. The exchange of the ICOS transmembrane domain for a CD8α transmembrane domain reduced CAR T cell efficacy to the level of 4-1BB-ICOS-ζ-CAR T cells, even in the absence of tonic signaling. As noted earlier, the ICOS transmembrane domain was shown to play a significant role in ICOS signaling [93] and was absent in CARs with proximal 4-1BB costimulation [35].

The hypothesis that the proximity of 4-1BB to the cell membrane may alter downstream signaling is in alignment with work from Gomes da Silva and colleagues, who reported 4-1BB-mediated tonic signaling in a second-generation CAR leading to the activation of the γ-retroviral long terminal repeat (LTR) promoter. Thereby, CAR surface expression was increased, which further exacerbated the tonic 4-1BB signal. Tonic 4-1BB signaling consequently promoted Fas-dependent AICD in CAR T cells [34]. However, when a CD28 domain was incorporated proximal to the 4-1BB domain to form a third-generation CAR, T cells demonstrated robust expansion and longer persistence in patients compared to a second-generation CAR with CD28 costimulation alone [113]. Thus, there is an increasing amount of evidence to support the hypothesis that the proximity of 4-1BB to the cell membrane may facilitate tonic signaling [35,38,113].

It remains to be seen whether third-generation CAR T cells will confer a clinical benefit compared to second-generation CAR T cells, and their successful implementation will most likely depend on furthering our understanding of how multiple costimulatory domains interact in order to optimize CAR signaling.

## 6. Transgene Delivery and Genome Editing

One central aspect of successful CAR T cell engineering is the method by which the CAR is introduced into the T cell. Both pre-clinically and clinically, this has primarily been achieved through the use of γ-retroviral or lentiviral vectors. In both cases, the transgene is integrated stably into the genome, which makes this approach suitable for the long-term genetic modification of cells [115]. One distinct advantage of lentiviral over γ-retroviral vectors is their ability to integrate into non-dividing cells [116]. Both γ-retroviruses and lentiviruses integrate semi-randomly within the host cell genome, thereby harboring the risk for insertional mutagenesis and dysregulation of genes adjacent to the integration site [117,118]. The risk for insertional oncogenesis is thought to be greater for certain γ-retroviral vectors, due to their propensity to integrate near the transcription start site of genes, as well as near proto-oncogenes [119,120]. Indeed, transgene integration in the proximity of proto-oncogenes, and the subsequent malignant transformation of modified hematopoietic stem cells (HSC) has been observed in clinical studies using γ-retroviral-mediated gene transfer to correct X-linked severe combined immunodeficiency (SCID) and Wiskott–Aldrich syndrome, respectively [121,122]. The risk for the insertional activation of oncogenes and consequential malignant transformation may be reduced but is not eliminated by the use of lentiviral vectors [123], with a high incidence of lentiviral vector-associated tumorigenesis having been observed in one in vivo model [124]. In fact, Maruggi and colleagues could show that retroviral genotoxicity is strongly influenced by the incorporation of viral promoter and enhancer regions within the retroviral vector design. The authors demonstrated that the ability of retroviral vectors to perturb gene expression in modified cells relies primarily on the activity of the regulatory elements used to drive transgene expression, rather than on the type of vector used [125]. Therefore, the development of self-inactivating vectors, through the deletion of enhancer elements from the viral LTR, allows for transgene expression via less active internal promoters, and thus may substantially reduce the risk of insertional gene dysregulation [125,126].

Importantly, to date, there have not been reports of CAR T cell therapy leading to the malignant transformation of transferred cells, and in some cases the near decade-long safety of γ-retrovirally modified T cells has been documented [127,128]. This suggests that the type of cell modified may also influence the risk for malignant transformation, and retroviral transduction of mature T cells seems comparably safe [127,128].

Transposon systems, such as Sleeping Beauty, constitute a non-viral method for gene transfer and present a cost-efficient alternative to the expensive production of good manufacturing practice (GMP)-compliant virus for clinical application. This “cut and paste” approach is used to stably integrate the transgene into the target cell genome, thereby also harboring the potential for insertional mutagenesis, however the anticipated risk for genotoxicity seems to be lower compared to retroviral vectors [129]. Furthermore, certain transposon systems are, at least in theory, capable of delivering large transgene cassettes, albeit at the cost of less efficient transposition [130]. This may be beneficial in attempts to equip CAR T cells with as many tools as possible to enhance their functional capacity against ever evolving cancer cells. Transposon systems have not yet been studied as extensively as conventional gene transfer methods, and their clinical standing compared to the more widely used retroviral vectors has yet to be determined.

Transient CAR expression may be achieved via ribonucleic acid (RNA)-electroporation, with virtually no risk for genotoxicity, since RNA does not integrate into the genome and is eventually fully degraded [131]. T cells can be modified to express a CAR for up to several days at high efficiencies, and RNA-electroporation may therefore present an attractive method to assess the on-target off-tumor toxicity of novel CARs [132,133]. The downside of this transient expression system is the rather rapid loss of the transgene, especially in proliferating cells, as the CAR-encoding RNA is not replicated during cell division. This may necessitate repeated infusions of CAR T cells to achieve a therapeutic effect, increasing the risk for an anti-CAR T cell immune response [63,132].

In conclusion, there are several viable options for CAR transgene delivery, all of which encompass unique advantages and risks. Due to the potentially severe consequences, it seems especially relevant to consider the risk for genotoxicity with regards to random transgene integration. The advent of new gene-editing tools such as clustered regularly interspaced short palindromic repeats (CRISPR)/Cas9 may allow for the simple and potentially safer “targeted insertion” of transgenes into T cells. One example of this was provided by Eyquem and colleagues, who utilized either Cas9 or transcription activator-like effector nuclease (TALEN) endonucleases in conjunction with an adeno-associated virus (AAV) vector repair matrix to specifically target an anti-CD19-CAR to the T cell receptor α constant (TRAC) or beta-2-microglobulin (B2M) locus in primary human T cells [112]. This approach demonstrated the feasibility of introducing a CAR in a desired genomic location, and mapping of integration sites revealed no off-target hotspots. However, reports of CRISPR/Cas9 leading to high-frequency off-target mutations in human cells exist, and should be considered when employing this technology [134]. As an alternative to a viral vector repair matrix, Roth and colleagues showed that linear double stranded deoxyribonucleic acid (dsDNA) can be co-electroporated with CRISPR/Cas9 ribonucleoprotein to mitigate dsDNA toxicity, which then serves as a homology-directed repair template, thereby completely bypassing the need for GMP-compatible production of viral vectors [135].

The ability to target specific sites within the genome may increase the safety of gene editing, as it offers the possibility to direct the CAR to proposed “genomic safe harbors” [136,137] which may be preferable sites for transgene integration to minimize genotoxicity and to optimize transgene function. Furthermore, the CAR may be targeted to certain genomic loci, with the intent to disrupt gene expression to increase CAR T cell potency. This was illustrated by a report from Fraietta and colleagues, who demonstrated that the progeny of a single CAR T cell clone displayed massive in vivo expansion and consequently led to complete remission of CLL in a 78-year-old patient, who had previously undergone CAR T cell therapy without success [138]. Subsequent analysis revealed that lentiviral vector integration had accidentally disrupted the methylcytosin dioxygenase TET2 gene on one allele. This led to the loss of the function of TET2 in CAR T cells, as the patient was found to harbor a hypomorphic mutation in his second allele. At the peak of the response, these TET2-deficient CAR T cells constituted 94% of the CD8^+^ CAR T cell repertoire. Furthermore, TET2-deficient CAR T cells predominantly displayed a central memory phenotype and could be detected in the patient’s blood four years after infusion. While TET2 is a tumor suppressor gene, there was no evidence of the malignant transformation of transferred cells. Experimental knockdown of TET2 recapitulated the phenotypic and functional changes observed in this patient’s CAR T cells, pointing towards the potential of TET2 disruption to enhance CAR T cell performance.

Additionally, gene-editing methods such as CRISPR/Cas9, TALENs and zinc finger nucleases (ZFNs) have been used to disrupt genes encoding for T cell suppressive receptors, thereby rendering T cells less susceptible to tumor-mediated immunosuppression [139,140,141,142]. Similarly, through the knockout of genes essential for the expression of endogenous TCR as well as human leukocyte antigen (HLA) molecules, these tools can facilitate the generation of allogeneic “universal” CAR T cells, amenable to patients independent of HLA matching by reducing the potential for graft-versus-host disease (GVHD) [139,143].

However, caution is advisable, as pitfalls in novel methods only ever become apparent after widespread use. In this regard, it has recently been shown that DNA double-strand breaks induced by CRISPR/Cas9 can elicit a p53-dependent DNA damage response in certain cell types [144,145]. In the context of adoptive cell therapy, the ill-considered use of this tool may therefore harbor the risk of selecting cells with a dysfunctional p53 pathway, which may lead to the increased genome instability of modified cells and thus could constitute a step towards tumorigenesis.

## 7. Promoter and Transgene Regulation

Another crucial consideration for effective CAR T cell design is how the transgene will be regulated once it is introduced into the T cell. Generally, this aspect of CAR design is strongly impacted by the type of transgene vector used. Gene expression after conventional γ-retroviral transduction is usually driven from enhancer and promoter elements in the retroviral LTR of the vector. In contrast, the self-inactivating design most commonly realized in lentiviral vectors, utilizes an internal promoter to drive transgene expression. Another complicating factor is that retrovirally inserted transgenes may be subject to silencing, resulting in variegated transgene expression [112,146]. Additionally, the number of vector copies integrated into the genome (insertion frequency) may influence the level of transgene expression and susceptibility to silencing [146,147,148,149]. The insertion frequency is strongly impacted by the multiplicity of infection (MOI) used during the transduction protocol, and is therefore a potential variable that may be altered [148]. All of these factors converge to determine the resulting level of CAR expression in the modified T cell. As noted earlier, the level of CAR expression is important to the extent that insufficient receptor density may decrease the sensitivity of CAR T cells for low-level antigen expressing tumor cells [52]. On the other hand, high-level CAR surface expression, driven by the elongation factor-1α (EF-1α) promoter, has been shown to facilitate antigen-independent tonic signaling in c-Met- and mesothelin-specific CAR T cells [36]. Tonic signaling resulted in constitutive secretion of high levels of cytokines and continuous proliferation of CAR T cells in the absence of exogenous cytokines or feeder cells. This “continuous” CAR T cell phenotype could be reverted to a “non-continuous” phenotype by replacing the EF-1α promoter for either a cytomegalovirus (CMV) promoter or a phosphoglycerate kinase 1 (PGK) promoter mutant, consequently reducing CAR surface expression. Importantly, this difference in CAR surface expression strongly impacted the ability of anti-c-Met CAR T cells to control tumor growth in a xenograft mouse model. When the expression of the anti-c-Met CAR was driven by the PGK100-promoter mutant, CAR T cells displayed greater in vivo persistence, enhanced anti-tumor efficacy and conferred a survival advantage compared to CAR T cells which utilized EF-1α to drive CAR expression.

Similarly, Gomes da Silva and colleagues reported that high anti-CD19-CAR expression driven by the LTR of the SFG γ-retroviral vector led to antigen-independent tonic 4-1BB signaling, which resulted in Fas-dependent apoptosis of CAR T cells [34]. Additionally, the authors could demonstrate the existence of a positive feedback loop dependent on the activation of the LTR promoter which led to a further increase in CAR expression. In contrast, when T cells were transduced with a self-inactivating lentiviral vector utilizing the EF-1α promoter, this deleterious feedback loop was interrupted leading to an overall reduction in CAR surface expression. This attenuated CAR expression normalized Fas and Fas ligand (Fas-L) expression on CAR T cells, and significantly reduced the frequency of cell death. Moreover, lentiviral CAR expression restored the proliferative capacity of T cells and promoted more efficient clearance of tumor cells in vitro. Finally, lentivirally-transduced anti-CD19-CAR T cells displayed superior anti-tumor activity in a xenograft mouse model compared to their γ-retrovirally transduced counterparts. This study not only underscores the crucial impact of CAR expression levels, but also forges an important link between the costimulatory domain and the choice of transgene promoter [34].

Interestingly, Eyquem and colleagues recently demonstrated that besides baseline CAR expression, the dynamic downregulation and re-expression kinetics following antigen exposure drastically impact the efficacy of CAR T cells [112]. This became apparent, when the authors targeted an anti-CD19-CAR to the TRAC locus, placing it under the control of the endogenous TCR promoter (TRAC-CAR). TRAC-CAR T cells outperformed both conventional retrovirally transduced CAR T cells (RV-CAR) as well as T cells with the CAR inserted in the B2M locus (B2M-CAR), in an in vivo mouse model. To investigate the impact of CAR expression on T cell performance, the authors generated T cells that expressed the CAR either from a randomly integrating γ-retroviral vector or under the control of different promoters from within two distinct genomic loci (TRAC, B2M). All of the promoter-locus combinations that conferred higher CAR expression than TRAC-CAR promoted tonic CAR signaling, in contrast to those providing lower expression. Furthermore, CAR T cells displayed distinct downregulation and re-expression kinetics of their receptors upon antigen encounter. While all CAR T cell groups downregulated CAR expression within 12 h of initial antigen encounter, TRAC-EF-1α, TRAC-LTR and RV-CAR T cells increased CAR expression within 24–48 h of antigen stimulation. In contrast, both TRAC and B2M-CAR T cells maintained CAR expression below baseline for 48 h after the initial downregulation of their receptors. Although the B2M-CAR responded similarly to antigen encounter as the TRAC-CAR, it did not perform as well in vivo. The authors speculate that this might be due to the lower basal expression level of B2M-CAR, which may be insufficient for effective CAR activity [112].

In conclusion, these reports highlight the complexity of CAR regulation and lay the groundwork for further investigation.

## 8. The Next Generation of CAR T Cells

**Figure 3:**
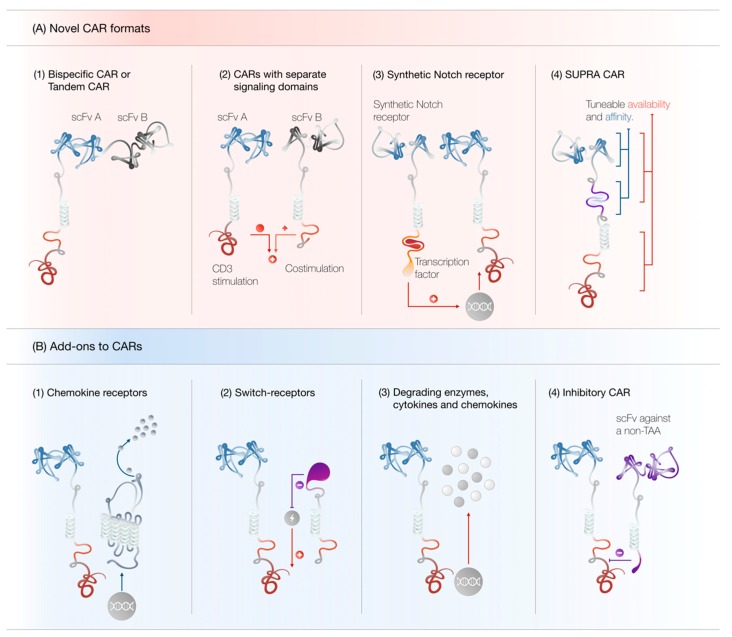
(**A**) Novel CAR formats. (1) Tandem CAR T cells can recognize either tumor antigen A or B and may prevent antigen escape. (2) CARs with separated signaling domains display attenuated off-tumor toxicity. (3) Synthetic Notch receptors can induce the transcription of another transgene, such as a conventional CAR, upon antigen recognition. (4) SUPRA CAR T cell activity may be tuned at multiple levels to ensure maximal effectiveness and minimal toxicity. (**B**) Add-ons to CARs. (1) CARs may be combined with chemokine receptors to increase CAR T cell trafficking to the tumor site. (2) Switch receptors can convert negative stimuli into positive ones. (3) CAR T cells can be engineered to secrete chemokines, cytokines and matrix-degrading enzymes to potentiate their anti-tumor efficacy. (4) Inhibitory CARs may be used to protect healthy tissue from CAR T cell-mediated destruction. Abbreviations: SUPRA CAR, split, universal and programmable CAR; TAA, tumor-associated antigen.

It is highly unlikely that the current format of CAR design will enable T cells to overcome the diverse obstacles presented by both tumor cells and the hostile tumor microenvironment. Therefore, several groups have devised innovative strategies in order to mitigate some of the intrinsic shortcomings of CAR T cells (see Figure 3). For instance, antigen loss and tumor escape may be prevented by simultaneously targeting more than one antigen on tumor cells. This can be achieved via the expression of multiple CARs with different specificities in one T cell [61,62,150]. Alternatively, a bispecific CAR (also termed tandem CAR) may be utilized, which incorporates two distinct antigen-recognition domains within one receptor. These receptors enable T cells to recognize tumor cells expressing either one of the antigens [151,152,153].

In an effort to increase T cell functionality and render the immunosuppressive tumor microenvironment permissive to an effective immune response, CAR T cells have been engineered to constitutively [154,155,156] or conditionally [157] secrete cytokines, such as IL-12, IL-15 and IL-18. In addition, dominant negative receptors can neutralize immunosuppressive ligands [158], whereas chimeric switch receptors may be used to convert inhibitory signals into T cell stimulatory signals [159,160,161,162,163]. The inability of tumor-specific T cells to effectively traffic and invade into the tumor site may be addressed through the concomitant expression of chemokine receptors [164,165,166] or enzymes, which degrade components of the extracellular matrix [167]. Furthermore, CAR T cells can be engineered to secrete chemokines to recruit the recipient’s endogenous immune system to potentiate their anti-tumor efficacy [168].

On the other hand, safety concerns regarding the toxicity of CAR T cells have incentivized the development of “off-switches” in the form of suicide genes, such as inducible-caspase-9 (iCASP9), herpes simplex virus thymidine kinase (HSV-TK) or truncated surface receptors, which permit the selective ablation of modified T cells through small molecules or antibodies, respectively (reviewed in [169,170]). Furthermore, the development of CAR T cells, where the CD3ζ and costimulatory domain are separated into two receptors targeting different tumor antigens, may mitigate on-target off-tumor toxicity by restricting the potent activation of CAR T cells to target cells expressing both antigens [171,172]. In addition, split CARs can be designed so that the antigen-binding receptor and downstream signaling domains only associate in the presence of a dimerizing small molecule, allowing for CAR T cell activity to be regulated via “remote control” [173]. Furthermore, the use of an inhibitory CAR (iCAR) can abrogate T cell activation in the presence of certain antigens and can be utilized to protect healthy tissue from CAR T cell-mediated destruction [174].

Another innovative approach to achieve greater tumor selectivity is the use of synthetic Notch receptors. As these receptors recognize one tumor-associated antigen, a transcription factor is cleaved from the intracellular domain and induces the transcription of another transgene, such as a conventional CAR, specific for a second tumor-associated antigen. Thus, synthetic Notch receptor CAR T cells only display effector functions against targets expressing both antigens [175]. Moreover, synthetic Notch receptors can confer T cells with the antigen-restricted ability to produce various cytokines, express death receptor ligands, secrete checkpoint blocking antibodies and differentiate into distinct T cell subsets [176].

A versatile approach to control T cell specificity and activation is the so-called split, universal and programmable CAR system (SUPRA-CAR) [177]. This platform utilizes leucine zippers to generate zip-CAR T cells, which may be redirected towards tumor antigens in conjunction with a zip-scFv. The zip-CAR T cell binds specifically to the zip-scFv, which in turn binds to an antigen expressed on tumor cells. Through the exchange of the zip-scFv specificity, CAR T cells may be redirected against multiple antigens at will, without the need to reengineer T cells. The magnitude of the SUPRA-CAR T cell response may be regulated via zip-scFv concentration or tuning of the scFv- or zipper-affinity for their targets. Additionally, zip-CARs may be constructed with the CD3ζ signal and costimulatory signal separated into two receptors. Thereby, the full activation of T cells is achieved only in the presence of multiple antigens. Furthermore, multiple zip-CARs may be utilized to differentially control the activation of different T cell subsets [177].

## 9. Conclusions

While CAR T cell therapy has come a long way and has achieved remarkable success in hematological malignancies, this therapeutic modality is still in its infancy and the underlying mechanisms which govern CAR T cell effectiveness are only gradually being elucidated. As CAR constructs become more complex, a thorough understanding of the impact of distinct domains would likely improve the rational design of CAR T cells to suit the specific needs of individual patients. The optimal choice of CAR domains will depend on a comprehensive understanding of the interaction between individual CAR components and the signaling cascades initiated through various intracellular domains. Owing to the complex nature of tumorigenesis, even optimized CARs may not suffice to overcome all the obstacles presented by various tumor entities. Therefore, we believe, that multiple modifications will have to be combined to compensate for some of the intrinsic shortcomings of CAR T cells and to meet the unique requirements posed by tumor cells and the tumor microenvironment. CAR T cell migration, infiltration and persistence will have to be optimized in order to achieve the best therapeutic outcome. To date, the lack of truly tumor-specific antigens is hampering the success of CAR T cell therapy in solid malignancies. In this regard, the combinatorial targeting of CAR T cells may allow for the specific destruction of tumor cells by integrating the presence or absence of multiple antigens to determine a CAR T cell’s response. Controlling CAR T cell activation and persistence is necessary to mitigate CAR T cell-mediated toxicities and restrict T cell effector functions to the tumor site. It is likely that novel gene-editing techniques herald a new era of CAR T cell engineering by potentiating CAR T cell efficacy and increasing the safety profile of gene-modified cell therapies. Since their conception three decades ago, CAR T cells have matured from an experimental treatment to a potent therapeutic option for patients with an otherwise poor prognosis. Nevertheless, there are still many obstacles to overcome on the long and winding road to successful cancer therapy and further research is needed to fully unleash the potential of CAR T cells.

## References

[1] Martin-Liberal J., Ochoa de Olza M., Hierro C., Gros A., Rodon J., Tabernero J. (2017). The expanding role of immunotherapy. Cancer Treat. Rev..

[2] Kobold S., Duewell P., Schnurr M., Subklewe M., Rothenfusser S., Endres S. (2015). Immunotherapy in tumors. Dtsch. Arztebl. Int..

[3] Duwell P., Heidegger S., Kobold S. (2019). Innate immune stimulation in cancer therapy. Hematol. Oncol. Clin. N. Am..

[4] Turcotte S., Gros A., Hogan K., Tran E., Hinrichs C.S., Wunderlich J.R., Dudley M.E., Rosenberg S.A. (2013). Phenotype and function of T cells infiltrating visceral metastases from gastrointestinal cancers and melanoma: Implications for adoptive cell transfer therapy. J. Immunol..

[5] Hall M., Liu H., Malafa M., Centeno B., Hodul P.J., Pimiento J., Pilon-Thomas S., Sarnaik A.A. (2016). Expansion of tumor-infiltrating lymphocytes (TIL) from human pancreatic tumors. J. Immunother. Cancer.

[6] Carretero R., Romero J.M., Ruiz-Cabello F., Maleno I., Rodriguez F., Camacho F.M., Real L.M., Garrido F., Cabrera T. (2008). Analysis of HLA class I expression in progressing and regressing metastatic melanoma lesions after immunotherapy. Immunogenetics.

[7] Del Campo A.B., Kyte J.A., Carretero J., Zinchencko S., Mendez R., Gonzalez-Aseguinolaza G., Ruiz-Cabello F., Aamdal S., Gaudernack G., Garrido F. (2014). Immune escape of cancer cells with beta2-microglobulin loss over the course of metastatic melanoma. Int. J. Cancer.

[8] Benmebarek M.R., Karches C.H., Cadilha B.L., Lesch S., Endres S., Kobold S. (2019). Killing mechanisms of chimeric antigen receptor (CAR) T cells. Int. J. Mol. Sci..

[9] Gross G., Waks T., Eshhar Z. (1989). Expression of immunoglobulin-T-cell receptor chimeric molecules as functional receptors with antibody-type specificity. Proc. Natl. Acad. Sci. USA.

[10] Eshhar Z., Waks T., Gross G., Schindler D.G. (1993). Specific activation and targeting of cytotoxic lymphocytes through chimeric single chains consisting of antibody-binding domains and the gamma or zeta subunits of the immunoglobulin and T-cell receptors. Proc. Natl. Acad. Sci. USA.

[11] Jensen M.C., Popplewell L., Cooper L.J., DiGiusto D., Kalos M., Ostberg J.R., Forman S.J. (2010). Antitransgene rejection responses contribute to attenuated persistence of adoptively transferred CD20/CD19-specific chimeric antigen receptor redirected T cells in humans. Biol. Blood Marrow Transplant..

[12] Till B.G., Jensen M.C., Wang J., Chen E.Y., Wood B.L., Greisman H.A., Qian X., James S.E., Raubitschek A., Forman S.J. (2008). Adoptive immunotherapy for indolent non-Hodgkin lymphoma and mantle cell lymphoma using genetically modified autologous CD20-specific T cells. Blood.

[13] Savoldo B., Ramos C.A., Liu E., Mims M.P., Keating M.J., Carrum G., Kamble R.T., Bollard C.M., Gee A.P., Mei Z. (2011). CD28 costimulation improves expansion and persistence of chimeric antigen receptor-modified T cells in lymphoma patients. J. Clin. Investig..

[14] Maude S.L., Frey N., Shaw P.A., Aplenc R., Barrett D.M., Bunin N.J., Chew A., Gonzalez V.E., Zheng Z., Lacey S.F. (2014). Chimeric antigen receptor T cells for sustained remissions in leukemia. N. Engl. J. Med..

[15] Kochenderfer J.N., Dudley M.E., Kassim S.H., Somerville R.P., Carpenter R.O., Stetler-Stevenson M., Yang J.C., Phan G.Q., Hughes M.S., Sherry R.M. (2015). Chemotherapy-refractory diffuse large B-cell lymphoma and indolent B-cell malignancies can be effectively treated with autologous T cells expressing an anti-CD19 chimeric antigen receptor. J. Clin. Oncol..

[16] Turtle C.J., Hay K.A., Hanafi L.A., Li D., Cherian S., Chen X., Wood B., Lozanski A., Byrd J.C., Heimfeld S. (2017). Durable molecular remissions in chronic lymphocytic leukemia treated with CD19-Specific chimeric antigen receptor-modified T cells after failure of Ibrutinib. J. Clin. Oncol..

[17] U.S. Food and Drug Administration KYMRIAH (tisagenlecleucel). https://www.fda.gov/biologicsbloodvaccines/cellulargenetherapyproducts/approvedproducts/ucm573706.htm.

[18] U.S. Food and Drug Administration YESCARTA (axicabtagene ciloleucel). https://www.fda.gov/biologicsbloodvaccines/cellulargenetherapyproducts/approvedproducts/ucm581222.htm.

[19] Porter D.L., Hwang W.T., Frey N.V., Lacey S.F., Shaw P.A., Loren A.W., Bagg A., Marcucci K.T., Shen A., Gonzalez V. (2015). Chimeric antigen receptor T cells persist and induce sustained remissions in relapsed refractory chronic lymphocytic leukemia. Sci. Transl. Med..

[20] Kochenderfer J.N., Dudley M.E., Carpenter R.O., Kassim S.H., Rose J.J., Telford W.G., Hakim F.T., Halverson D.C., Fowler D.H., Hardy N.M. (2013). Donor-derived CD19-targeted T cells cause regression of malignancy persisting after allogeneic hematopoietic stem cell transplantation. Blood.

[21] Lee D.W., Kochenderfer J.N., Stetler-Stevenson M., Cui Y.K., Delbrook C., Feldman S.A., Fry T.J., Orentas R., Sabatino M., Shah N.N. (2015). T cells expressing CD19 chimeric antigen receptors for acute lymphoblastic leukaemia in children and young adults: A phase 1 dose-escalation trial. Lancet.

[22] Fitzgerald J.C., Weiss S.L., Maude S.L., Barrett D.M., Lacey S.F., Melenhorst J.J., Shaw P., Berg R.A., June C.H., Porter D.L. (2017). Cytokine release syndrome after chimeric antigen receptor T cell therapy for acute lymphoblastic leukemia. Crit. Care Med..

[23] Gardner R.A., Finney O., Annesley C., Brakke H., Summers C., Leger K., Bleakley M., Brown C., Mgebroff S., Kelly-Spratt K.S. (2017). Intent-to-treat leukemia remission by CD19 CAR T cells of defined formulation and dose in children and young adults. Blood.

[24] Park J.H., Riviere I., Gonen M., Wang X., Senechal B., Curran K.J., Sauter C., Wang Y., Santomasso B., Mead E. (2018). Long-term follow-up of CD19 CAR therapy in acute lymphoblastic leukemia. N. Engl. J. Med..

[25] Grupp S.A., Kalos M., Barrett D., Aplenc R., Porter D.L., Rheingold S.R., Teachey D.T., Chew A., Hauck B., Wright J.F. (2013). Chimeric antigen receptor-modified T cells for acute lymphoid leukemia. N. Engl. J. Med..

[26] Morgan R.A., Yang J.C., Kitano M., Dudley M.E., Laurencot C.M., Rosenberg S.A. (2010). Case report of a serious adverse event following the administration of T cells transduced with a chimeric antigen receptor recognizing ERBB2. Mol. Ther..

[27] Lamers C.H., Sleijfer S., Vulto A.G., Kruit W.H., Kliffen M., Debets R., Gratama J.W., Stoter G., Oosterwijk E. (2006). Treatment of metastatic renal cell carcinoma with autologous T-lymphocytes genetically retargeted against carbonic anhydrase IX: First clinical experience. J. Clin. Oncol..

[28] Parkhurst M.R., Yang J.C., Langan R.C., Dudley M.E., Nathan D.A., Feldman S.A., Davis J.L., Morgan R.A., Merino M.J., Sherry R.M. (2011). T cells targeting carcinoembryonic antigen can mediate regression of metastatic colorectal cancer but induce severe transient colitis. Mol. Ther..

[29] Maude S.L., Laetsch T.W., Buechner J., Rives S., Boyer M., Bittencourt H., Bader P., Verneris M.R., Stefanski H.E., Myers G.D. (2018). Tisagenlecleucel in children and young adults with B-cell lymphoblastic leukemia. N. Engl. J. Med..

[30] Tokarew N., Ogonek J., Endres S., von Bergwelt-Baildon M., Kobold S. (2019). Teaching an old dog new tricks: Next-generation CAR T cells. Br. J. Cancer.

[31] Loureiro Cadilha B., Dorman K., Rataj F., Endres S., Kobold S. (2017). Enabling T cell recruitment to tumours as a strategy for improving adoptive T cell therapy. Eur. Oncol. Haematol..

[32] Zhang J., Endres S., Kobold S. (2019). Enhancing tumor T cell infiltration to enable cancer immunotherapy. Immunotherapy.

[33] Long A.H., Haso W.M., Shern J.F., Wanhainen K.M., Murgai M., Ingaramo M., Smith J.P., Walker A.J., Kohler M.E., Venkateshwara V.R. (2015). 4-1BB costimulation ameliorates T cell exhaustion induced by tonic signaling of chimeric antigen receptors. Nat. Med..

[34] Gomes-Silva D., Mukherjee M., Srinivasan M., Krenciute G., Dakhova O., Zheng Y., Cabral J.M.S., Rooney C.M., Orange J.S., Brenner M.K. (2017). Tonic 4-1BB costimulation in chimeric antigen receptors impedes T cell survival and Is vector-dependent. Cell Rep..

[35] Guedan S., Posey A.D., Shaw C., Wing A., Da T., Patel P.R., McGettigan S.E., Casado-Medrano V., Kawalekar O.U., Uribe-Herranz M. (2018). Enhancing CAR T cell persistence through ICOS and 4-1BB costimulation. JCI Insight.

[36] Frigault M.J., Lee J., Basil M.C., Carpenito C., Motohashi S., Scholler J., Kawalekar O.U., Guedan S., McGettigan S.E., Posey A.D. (2015). Identification of chimeric antigen receptors that mediate constitutive or inducible proliferation of T cells. Cancer Immunol. Res..

[37] Watanabe N., Bajgain P., Sukumaran S., Ansari S., Heslop H.E., Rooney C.M., Brenner M.K., Leen A.M., Vera J.F. (2016). Fine-tuning the CAR spacer improves T-cell potency. Oncoimmunology.

[38] Milone M.C., Fish J.D., Carpenito C., Carroll R.G., Binder G.K., Teachey D., Samanta M., Lakhal M., Gloss B., Danet-Desnoyers G. (2009). Chimeric receptors containing CD137 signal transduction domains mediate enhanced survival of T cells and increased antileukemic efficacy in vivo. Mol. Ther..

[39] Song D.G., Ye Q., Carpenito C., Poussin M., Wang L.P., Ji C., Figini M., June C.H., Coukos G., Powell D.J. (2011). In vivo persistence, tumor localization, and antitumor activity of CAR-engineered T cells is enhanced by costimulatory signaling through CD137 (4-1BB). Cancer Res..

[40] Bird R.E., Hardman K.D., Jacobson J.W., Johnson S., Kaufman B.M., Lee S.M., Lee T., Pope S.H., Riordan G.S., Whitlow M. (1988). Single-chain antigen-binding proteins. Science.

[41] Huston J.S., Levinson D., Mudgett-Hunter M., Tai M.S., Novotny J., Margolies M.N., Ridge R.J., Bruccoleri R.E., Haber E., Crea R. (1988). Protein engineering of antibody binding sites: Recovery of specific activity in an anti-digoxin single-chain Fv analogue produced in Escherichia coli. Proc. Natl. Acad. Sci. USA.

[42] Burns W.R., Zhao Y., Frankel T.L., Hinrichs C.S., Zheng Z., Xu H., Feldman S.A., Ferrone S., Rosenberg S.A., Morgan R.A. (2010). A high molecular weight melanoma-associated antigen-specific chimeric antigen receptor redirects lymphocytes to target human melanomas. Cancer Res..

[43] Richman S.A., Nunez-Cruz S., Moghimi B., Li L.Z., Gershenson Z.T., Mourelatos Z., Barrett D.M., Grupp S.A., Milone M.C. (2018). High-affinity GD2-specific CAR T cells induce fatal encephalitis in a preclinical neuroblastoma model. Cancer Immunol. Res..

[44] Argos P. (1990). An investigation of oligopeptides linking domains in protein tertiary structures and possible candidates for general gene fusion. J. Mol. Biol..

[45] Chen X., Zaro J.L., Shen W.C. (2013). Fusion protein linkers: Property, design and functionality. Adv. Drug Deliv. Rev..

[46] Dolezal O., Pearce L.A., Lawrence L.J., McCoy A.J., Hudson P.J., Kortt A.A. (2000). ScFv multimers of the anti-neuraminidase antibody NC10: Shortening of the linker in single-chain Fv fragment assembled in V(L) to V(H) orientation drives the formation of dimers, trimers, tetramers and higher molecular mass multimers. Protein Eng..

[47] Whitlow M., Filpula D., Rollence M.L., Feng S.-L., Wood J.F. (1994). Multivalent Fvs: Characterization of single-chain Fv oligomers and preparation of a bispecific Fv. Protein Eng..

[48] Rafiq S., Purdon T.J., Daniyan A.F., Koneru M., Dao T., Liu C., Scheinberg D.A., Brentjens R.J. (2017). Optimized T-cell receptor-mimic chimeric antigen receptor T cells directed toward the intracellular Wilms Tumor 1 antigen. Leukemia.

[49] Hege K.M., Bergsland E.K., Fisher G.A., Nemunaitis J.J., Warren R.S., McArthur J.G., Lin A.A., Schlom J., June C.H., Sherwin S.A. (2017). Safety, tumor trafficking and immunogenicity of chimeric antigen receptor (CAR)-T cells specific for TAG-72 in colorectal cancer. J. Immunother. Cancer.

[50] Kunkele A., Johnson A.J., Rolczynski L.S., Chang C.A., Hoglund V., Kelly-Spratt K.S., Jensen M.C. (2015). Functional tuning of CARs reveals signaling threshold above which CD8^+^ CTL antitumor potency is attenuated due to cell Fas-FasL-dependent AICD. Cancer Immunol. Res..

[51] Courtney A.H., Lo W.L., Weiss A. (2018). TCR Signaling: Mechanisms of initiation and propagation. Trends Biochem. Sci..

[52] Liu X., Jiang S., Fang C., Yang S., Olalere D., Pequignot E.C., Cogdill A.P., Li N., Ramones M., Granda B. (2015). Affinity-tuned ErbB2 or EGFR chimeric antigen receptor T cells exhibit an increased therapeutic index against tumors in mice. Cancer Res..

[53] Hudecek M., Lupo-Stanghellini M.T., Kosasih P.L., Sommermeyer D., Jensen M.C., Rader C., Riddell S.R. (2013). Receptor affinity and extracellular domain modifications affect tumor recognition by ROR1-specific chimeric antigen receptor T cells. Clin. Cancer Res..

[54] Lynn R.C., Feng Y., Schutsky K., Poussin M., Kalota A., Dimitrov D.S., Powell D.J. (2016). High-affinity FRbeta-specific CAR T cells eradicate AML and normal myeloid lineage without HSC toxicity. Leukemia.

[55] Chmielewski M., Hombach A., Heuser C., Adams G.P., Abken H. (2004). T cell activation by antibody-like immunoreceptors: Increase in affinity of the single-chain fragment domain above threshold does not increase T cell activation against antigen-positive target cells but decreases selectivity. J. Immunol..

[56] Caruso H.G., Hurton L.V., Najjar A., Rushworth D., Ang S., Olivares S., Mi T., Switzer K., Singh H., Huls H. (2015). Tuning sensitivity of CAR to EGFR density limits recognition of normal tissue while maintaining potent antitumor activity. Cancer Res..

[57] Song D.-G., Ye Q., Poussin M., Liu L., Figini M., Powell D.J. (2015). A fully human chimeric antigen receptor with potent activity against cancer cells but reduced risk for off-tumor toxicity. Oncotarget.

[58] Posey A.D., Schwab R.D., Boesteanu A.C., Steentoft C., Mandel U., Engels B., Stone J.D., Madsen T.D., Schreiber K., Haines K.M. (2016). Engineered CAR T cells targeting the cancer-associated Tn-glycoform of the membrane mucin MUC1 control adenocarcinoma. Immunity.

[59] Mount C.W., Majzner R.G., Sundaresh S., Arnold E.P., Kadapakkam M., Haile S., Labanieh L., Hulleman E., Woo P.J., Rietberg S.P. (2018). Potent antitumor efficacy of anti-GD2 CAR T cells in H3-K27M^+^ diffuse midline gliomas. Nat. Med..

[60] Maus M.V., Plotkin J., Jakka G., Stewart-Jones G., Riviere I., Merghoub T., Wolchok J., Renner C., Sadelain M. (2016). An MHC-restricted antibody-based chimeric antigen receptor requires TCR-like affinity to maintain antigen specificity. Mol. Ther. Oncolytics.

[61] Ruella M., Barrett D.M., Kenderian S.S., Shestova O., Hofmann T.J., Perazzelli J., Klichinsky M., Aikawa V., Nazimuddin F., Kozlowski M. (2016). Dual CD19 and CD123 targeting prevents antigen-loss relapses after CD19-directed immunotherapies. J. Clin. Investig..

[62] Bielamowicz K., Fousek K., Byrd T.T., Samaha H., Mukherjee M., Aware N., Wu M.F., Orange J.S., Sumazin P., Man T.K. (2018). Trivalent CAR T cells overcome interpatient antigenic variability in glioblastoma. Neuro Oncol..

[63] Maus M.V., Haas A.R., Beatty G.L., Albelda S.M., Levine B.L., Liu X., Zhao Y., Kalos M., June C.H. (2013). T cells expressing chimeric antigen receptors can cause anaphylaxis in humans. Cancer Immunol. Res..

[64] Turtle C.J., Hanafi L.A., Berger C., Gooley T.A., Cherian S., Hudecek M., Sommermeyer D., Melville K., Pender B., Budiarto T.M. (2016). CD19 CAR-T cells of defined CD4^+^:CD8^+^ composition in adult B cell ALL patients. J. Clin. Investig..

[65] Sun M., Shi H., Liu C., Liu J., Liu X., Sun Y. (2014). Construction and evaluation of a novel humanized HER2-specific chimeric receptor. Breast Cancer Res..

[66] Maude S.L., Barrett D.M., Ambrose D.E., Rheingold S.R., Aplenc R., Teachey D.T., Callahan C., Barker C.S., Mudambi M., Shaw P.A. (2015). Efficacy and safety of humanized chimeric antigen receptor (CAR)-modified T cells targeting CD19 in children with relapsed/refractory ALL. Blood.

[67] Alabanza L., Pegues M., Geldres C., Shi V., Wiltzius J.J.W., Sievers S.A., Yang S., Kochenderfer J.N. (2017). Function of novel anti-CD19 chimeric antigen receptors with human variable regions is affected by hinge and transmembrane domains. Mol. Ther..

[68] Brown C.E., Badie B., Barish M.E., Weng L., Ostberg J.R., Chang W.C., Naranjo A., Starr R., Wagner J., Wright C. (2015). Bioactivity and safety of IL13Ralpha2-redirected chimeric antigen receptor CD8^+^ T cells in patients with recurrent glioblastoma. Clin. Cancer Res..

[69] Baumeister S.H., Murad J., Werner L., Daley H., Trebeden-Negre H., Gicobi J.K., Schmucker A., Reder J., Sentman C.L., Gilham D.E. (2019). Phase I trial of autologous CAR T cells targeting NKG2D ligands in patients with AML/MDS and multiple myeloma. Cancer Immunol. Res..

[70] De Munter S., Ingels J., Goetgeluk G., Bonte S., Pille M., Weening K., Kerre T., Abken H., Vandekerckhove B. (2018). Nanobody based dual specific CARs. Int. J. Mol. Sci..

[71] Rataj F., Jacobi S.J., Stoiber S., Asang F., Ogonek J., Tokarew N., Cadilha B.L., van Puijenbroek E., Heise C., Duewell P. (2019). High-affinity CD16-polymorphism and Fc-engineered antibodies enable activity of CD16-chimeric antigen receptor-modified T cells for cancer therapy. Br. J. Cancer.

[72] Murad J.M., Graber D.J., Sentman C.L. (2018). Advances in the use of natural receptor- or ligand-based chimeric antigen receptors (CARs) in haematologic malignancies. Best Pract. Res. Clin. Haematol..

[73] Darowski D., Kobold S., Jost C., Klein C. (2019). Combining the best of two worlds: Highly flexible chimeric antigen receptor adaptor molecules (CAR-adaptors) for the recruitment of chimeric antigen receptor T cells. MAbs.

[74] James S.E., Greenberg P.D., Jensen M.C., Lin Y., Wang J., Till B.G., Raubitschek A.A., Forman S.J., Press O.W. (2008). Antigen sensitivity of CD22-specific chimeric TCR is modulated by target epitope distance from the cell membrane. J. Immunol..

[75] Hombach A.A., Schildgen V., Heuser C., Finnern R., Gilham D.E., Abken H. (2007). T cell activation by antibody-like immunoreceptors: The position of the binding epitope within the target molecule determines the efficiency of activation of redirected T cells. J. Immunol..

[76] Haso W., Lee D.W., Shah N.N., Stetler-Stevenson M., Yuan C.M., Pastan I.H., Dimitrov D.S., Morgan R.A., FitzGerald D.J., Barrett D.M. (2013). Anti-CD22-chimeric antigen receptors targeting B-cell precursor acute lymphoblastic leukemia. Blood.

[77] Srivastava S., Riddell S.R. (2015). Engineering CAR-T cells: Design concepts. Trends Immunol..

[78] James J.R., Vale R.D. (2012). Biophysical mechanism of T-cell receptor triggering in a reconstituted system. Nature.

[79] Guest R.D., Hawkins R.E., Kirillova N., Cheadle E.J., Arnold J., O’Neill A., Irlam J., Chester K.A., Kemshead J.T., Shaw D.M. (2005). The role of extracellular spacer regions in the optimal design of chimeric immune receptors: Evaluation of four different scFvs and antigens. J. Immunother..

[80] Hudecek M., Sommermeyer D., Kosasih P.L., Silva-Benedict A., Liu L., Rader C., Jensen M.C., Riddell S.R. (2015). The nonsignaling extracellular spacer domain of chimeric antigen receptors is decisive for in vivo antitumor activity. Cancer Immunol. Res..

[81] Krenciute G., Krebs S., Torres D., Wu M.F., Liu H., Dotti G., Li X.N., Lesniak M.S., Balyasnikova I.V., Gottschalk S. (2016). Characterization and functional analysis of scFv-based chimeric antigen receptors to redirect T cells to IL13Ralpha2-positive glioma. Mol. Ther..

[82] Wilkie S., Picco G., Foster J., Davies D.M., Julien S., Cooper L., Arif S., Mather S.J., Taylor-Papadimitriou J., Burchell J.M. (2008). Retargeting of human T cells to tumor-associated MUC1: The evolution of a chimeric antigen receptor. J. Immunol..

[83] Hombach A., Hombach A.A., Abken H. (2010). Adoptive immunotherapy with genetically engineered T cells: Modification of the IgG1 Fc ‘spacer’ domain in the extracellular moiety of chimeric antigen receptors avoids ‘off-target’ activation and unintended initiation of an innate immune response. Gene Ther..

[84] Almasbak H., Walseng E., Kristian A., Myhre M.R., Suso E.M., Munthe L.A., Andersen J.T., Wang M.Y., Kvalheim G., Gaudernack G. (2015). Inclusion of an IgG1-Fc spacer abrogates efficacy of CD19 CAR T cells in a xenograft mouse model. Gene Ther..

[85] Jonnalagadda M., Mardiros A., Urak R., Wang X., Hoffman L.J., Bernanke A., Chang W.C., Bretzlaff W., Starr R., Priceman S. (2015). Chimeric antigen receptors with mutated IgG4 Fc spacer avoid fc receptor binding and improve T cell persistence and antitumor efficacy. Mol. Ther..

[86] Till B.G., Jensen M.C., Wang J., Qian X., Gopal A.K., Maloney D.G., Lindgren C.G., Lin Y., Pagel J.M., Budde L.E. (2012). CD20-specific adoptive immunotherapy for lymphoma using a chimeric antigen receptor with both CD28 and 4-1BB domains: Pilot clinical trial results. Blood.

[87] Gargett T., Yu W., Dotti G., Yvon E.S., Christo S.N., Hayball J.D., Lewis I.D., Brenner M.K., Brown M.P. (2016). GD2-specific CAR T cells undergo potent activation and deletion following antigen encounter but can be protected from activation-induced cell death by PD-1 blockade. Mol. Ther..

[88] Brown C.E., Alizadeh D., Starr R., Weng L., Wagner J.R., Naranjo A., Ostberg J.R., Blanchard M.S., Kilpatrick J., Simpson J. (2016). Regression of glioblastoma after chimeric antigen receptor T-cell therapy. N. Engl. J. Med..

[89] Kochenderfer J.N., Wilson W.H., Janik J.E., Dudley M.E., Stetler-Stevenson M., Feldman S.A., Maric I., Raffeld M., Nathan D.-A.N., Lanier B.J. (2010). Eradication of B-lineage cells and regression of lymphoma in a patient treated with autologous T cells genetically engineered to recognize CD19. Blood.

[90] Morin S.O., Giroux V., Favre C., Bechah Y., Auphan-Anezin N., Roncagalli R., Mège J.-L., Olive D., Malissen M., Nunès J.A. (2015). In the absence of its cytosolic domain, the CD28 molecule still contributes to T cell activation. Cell. Mol. Life Sci..

[91] Zhang T., Wu M.R., Sentman C.L. (2012). An NKp30-based chimeric antigen receptor promotes T cell effector functions and antitumor efficacy in vivo. J. Immunol..

[92] Bridgeman J.S., Hawkins R.E., Bagley S., Blaylock M., Holland M., Gilham D.E. (2010). The optimal antigen response of chimeric antigen receptors harboring the CD3zeta transmembrane domain is dependent upon incorporation of the receptor into the endogenous TCR/CD3 complex. J. Immunol..

[93] Wan Z., Shao X., Ji X., Dong L., Wei J., Xiong Z., Liu W., Qi H. (2018). Transmembrane domain-mediated Lck association underlies bystander and costimulatory ICOS signaling. Cell. Mol. Immunol..

[94] van der Stegen S.J., Hamieh M., Sadelain M. (2015). The pharmacology of second-generation chimeric antigen receptors. Nat. Rev. Drug Discov..

[95] Imai C., Mihara K., Andreansky M., Nicholson I.C., Pui C.H., Geiger T.L., Campana D. (2004). Chimeric receptors with 4-1BB signaling capacity provoke potent cytotoxicity against acute lymphoblastic leukemia. Leukemia.

[96] Pule M.A., Straathof K.C., Dotti G., Heslop H.E., Rooney C.M., Brenner M.K. (2005). A chimeric T cell antigen receptor that augments cytokine release and supports clonal expansion of primary human T cells. Mol. Ther..

[97] Carpenito C., Milone M.C., Hassan R., Simonet J.C., Lakhal M., Suhoski M.M., Varela-Rohena A., Haines K.M., Heitjan D.F., Albelda S.M. (2009). Control of large, established tumor xenografts with genetically retargeted human T cells containing CD28 and CD137 domains. Proc. Natl. Acad. Sci. USA.

[98] Zhao Z., Condomines M., van der Stegen S.J.C., Perna F., Kloss C.C., Gunset G., Plotkin J., Sadelain M. (2015). Structural design of engineered costimulation determines tumor rejection kinetics and persistence of CAR T cells. Cancer Cell.

[99] Kawalekar O.U., O’Connor R.S., Fraietta J.A., Guo L., McGettigan S.E., Posey A.D., Patel P.R., Guedan S., Scholler J., Keith B. (2016). Distinct signaling of coreceptors regulates specific metabolism pathways and impacts memory development in CAR T cells. Immunity.

[100] Salter A.I., Ivey R.G., Kennedy J.J., Voillet V., Rajan A., Alderman E.J., Voytovich U.J., Lin C., Sommermeyer D., Liu L. (2018). Phosphoproteomic analysis of chimeric antigen receptor signaling reveals kinetic and quantitative differences that affect cell function. Sci. Signal..

[101] Davila M.L., Riviere I., Wang X., Bartido S., Park J., Curran K., Chung S.S., Stefanski J., Borquez-Ojeda O., Olszewska M. (2014). Efficacy and toxicity management of 19-28z CAR T cell therapy in B cell acute lymphoblastic leukemia. Sci. Transl. Med..

[102] Brentjens R.J., Davila M.L., Riviere I., Park J., Wang X., Cowell L.G., Bartido S., Stefanski J., Taylor C., Olszewska M. (2013). CD19-targeted T cells rapidly induce molecular remissions in adults with chemotherapy-refractory acute lymphoblastic leukemia. Sci. Transl. Med..

[103] Fraietta J.A., Lacey S.F., Orlando E.J., Pruteanu-Malinici I., Gohil M., Lundh S., Boesteanu A.C., Wang Y., O’Connor R.S., Hwang W.T. (2018). Determinants of response and resistance to CD19 chimeric antigen receptor (CAR) T cell therapy of chronic lymphocytic leukemia. Nat. Med..

[104] Song D.G., Ye Q., Poussin M., Harms G.M., Figini M., Powell D.J. (2012). CD27 costimulation augments the survival and antitumor activity of redirected human T cells in vivo. Blood.

[105] Guedan S., Chen X., Madar A., Carpenito C., McGettigan S.E., Frigault M.J., Lee J., Posey A.D., Scholler J., Scholler N. (2014). ICOS-based chimeric antigen receptors program bipolar TH17/TH1 cells. Blood.

[106] Ahmed N., Brawley V., Hegde M., Bielamowicz K., Kalra M., Landi D., Robertson C., Gray T.L., Diouf O., Wakefield A. (2017). HER2-specific chimeric antigen receptor-modified virus-specific T cells for progressive glioblastoma: A phase 1 dose-escalation trial. JAMA Oncol..

[107] Pule M.A., Savoldo B., Myers G.D., Rossig C., Russell H.V., Dotti G., Huls M.H., Liu E., Gee A.P., Mei Z. (2008). Virus-specific T cells engineered to coexpress tumor-specific receptors: Persistence and antitumor activity in individuals with neuroblastoma. Nat. Med..

[108] Yang Y., Kohler M.E., Chien C.D., Sauter C.T., Jacoby E., Yan C., Hu Y., Wanhainen K., Qin H., Fry T.J. (2017). TCR engagement negatively affects CD8 but not CD4 CAR T cell expansion and leukemic clearance. Sci. Transl. Med..

[109] Zhong X.S., Matsushita M., Plotkin J., Riviere I., Sadelain M. (2010). Chimeric antigen receptors combining 4-1BB and CD28 signaling domains augment PI3kinase/AKT/Bcl-XL activation and CD8^+^ T cell-mediated tumor eradication. Mol. Ther..

[110] Yi Z., Prinzing B.L., Cao F., Gottschalk S., Krenciute G. (2018). Optimizing EphA2-CAR T cells for the adoptive immunotherapy of glioma. Mol. Ther. Methods Clin. Dev..

[111] Abate-Daga D., Lagisetty K.H., Tran E., Zheng Z., Gattinoni L., Yu Z., Burns W.R., Miermont A.M., Teper Y., Rudloff U. (2014). A novel chimeric antigen receptor against prostate stem cell antigen mediates tumor destruction in a humanized mouse model of pancreatic cancer. Hum. Gene Ther..

[112] Eyquem J., Mansilla-Soto J., Giavridis T., van der Stegen S.J., Hamieh M., Cunanan K.M., Odak A., Gonen M., Sadelain M. (2017). Targeting a CAR to the TRAC locus with CRISPR/Cas9 enhances tumour rejection. Nature.

[113] Gomes da Silva D., Mukherjee M., Madhuwanti S., Dakhova O., Liu H., Grilley B., Gee A.P., Neelapu S.S., Rooney C.M., Heslop H.E. (2017). Direct comparison of in vivo fate of second and third-generation CD19-specific chimeric antigen receptor (CAR)-T cells in patients with B-cell Non-Hodgkin lymphoma (B-NHL): Reversal of toxicity from tonic signaling. Biol. Blood Marrow Transplant..

[114] Ramello M.C., Benzaid I., Kuenzi B.M., Lienlaf-Moreno M., Kandell W.M., Santiago D.N., Pabon-Saldana M., Darville L., Fang B., Rix U. (2019). An immunoproteomic approach to characterize the CAR interactome and signalosome. Sci. Signal..

[115] Thomas C.E., Ehrhardt A., Kay M.A. (2003). Progress and problems with the use of viral vectors for gene therapy. Nat. Rev. Genet..

[116] Lewis P.F., Emerman M. (1994). Passage through mitosis is required for oncoretroviruses but not for the human immunodeficiency virus. J. Virol..

[117] Schroder A.R., Shinn P., Chen H., Berry C., Ecker J.R., Bushman F. (2002). HIV-1 integration in the human genome favors active genes and local hotspots. Cell.

[118] Wu X., Li Y., Crise B., Burgess S.M. (2003). Transcription start regions in the human genome are favored targets for MLV integration. Science.

[119] Cattoglio C., Facchini G., Sartori D., Antonelli A., Miccio A., Cassani B., Schmidt M., von Kalle C., Howe S., Thrasher A.J. (2007). Hot spots of retroviral integration in human CD34^+^ hematopoietic cells. Blood.

[120] Hematti P., Hong B.K., Ferguson C., Adler R., Hanawa H., Sellers S., Holt I.E., Eckfeldt C.E., Sharma Y., Schmidt M. (2004). Distinct genomic integration of MLV and SIV vectors in primate hematopoietic stem and progenitor cells. PLoS Biol..

[121] Hacein-Bey-Abina S., Garrigue A., Wang G.P., Soulier J., Lim A., Morillon E., Clappier E., Caccavelli L., Delabesse E., Beldjord K. (2008). Insertional oncogenesis in 4 patients after retrovirus-mediated gene therapy of SCID-X1. J. Clin. Investig..

[122] Braun C.J., Boztug K., Paruzynski A., Witzel M., Schwarzer A., Rothe M., Modlich U., Beier R., Gohring G., Steinemann D. (2014). Gene therapy for Wiskott-Aldrich syndrome--long-term efficacy and genotoxicity. Sci. Transl. Med..

[123] Cavazzana-Calvo M., Payen E., Negre O., Wang G., Hehir K., Fusil F., Down J., Denaro M., Brady T., Westerman K. (2010). Transfusion independence and HMGA2 activation after gene therapy of human beta-thalassaemia. Nature.

[124] Themis M., Waddington S.N., Schmidt M., von Kalle C., Wang Y., Al-Allaf F., Gregory L.G., Nivsarkar M., Themis M., Holder M.V. (2005). Oncogenesis following delivery of a nonprimate lentiviral gene therapy vector to fetal and neonatal mice. Mol. Ther..

[125] Maruggi G., Porcellini S., Facchini G., Perna S.K., Cattoglio C., Sartori D., Ambrosi A., Schambach A., Baum C., Bonini C. (2009). Transcriptional enhancers induce insertional gene deregulation independently from the vector type and design. Mol. Ther..

[126] Zychlinski D., Schambach A., Modlich U., Maetzig T., Meyer J., Grassman E., Mishra A., Baum C. (2008). Physiological promoters reduce the genotoxic risk of integrating gene vectors. Mol. Ther..

[127] Scholler J., Brady T.L., Binder-Scholl G., Hwang W.T., Plesa G., Hege K.M., Vogel A.N., Kalos M., Riley J.L., Deeks S.G. (2012). Decade-long safety and function of retroviral-modified chimeric antigen receptor T cells. Sci. Transl. Med..

[128] Recchia A., Bonini C., Magnani Z., Urbinati F., Sartori D., Muraro S., Tagliafico E., Bondanza A., Stanghellini M.T., Bernardi M. (2006). Retroviral vector integration deregulates gene expression but has no consequence on the biology and function of transplanted T cells. Proc. Natl. Acad. Sci. USA.

[129] Monjezi R., Miskey C., Gogishvili T., Schleef M., Schmeer M., Einsele H., Ivics Z., Hudecek M. (2017). Enhanced CAR T-cell engineering using non-viral Sleeping Beauty transposition from minicircle vectors. Leukemia.

[130] Turchiano G., Latella M.C., Gogol-Doring A., Cattoglio C., Mavilio F., Izsvak Z., Ivics Z., Recchia A. (2014). Genomic analysis of Sleeping Beauty transposon integration in human somatic cells. PLoS ONE.

[131] Foster J.B., Barrett D.M., Karikó K. (2019). The emerging role of in vitro-transcribed mRNA in adoptive T cell immunotherapy. Mol. Ther..

[132] Beatty G.L., Haas A.R., Maus M.V., Torigian D.A., Soulen M.C., Plesa G., Chew A., Zhao Y., Levine B.L., Albelda S.M. (2014). Mesothelin-specific chimeric antigen receptor mRNA-engineered T cells induce anti-tumor activity in solid malignancies. Cancer Immunol. Res..

[133] Caruso H.G., Torikai H., Zhang L., Maiti S., Dai J., Do K.A., Singh H., Huls H., Lee D.A., Champlin R.E. (2016). Redirecting T-cell specificity to EGFR using mRNA to self-limit expression of chimeric antigen receptor. J. Immunother..

[134] Fu Y., Foden J.A., Khayter C., Maeder M.L., Reyon D., Joung J.K., Sander J.D. (2013). High-frequency off-target mutagenesis induced by CRISPR-Cas nucleases in human cells. Nat. Biotechnol..

[135] Roth T.L., Puig-Saus C., Yu R., Shifrut E., Carnevale J., Li P.J., Hiatt J., Saco J., Krystofinski P., Li H. (2018). Reprogramming human T cell function and specificity with non-viral genome targeting. Nature.

[136] Sadelain M., Papapetrou E.P., Bushman F.D. (2011). Safe harbours for the integration of new DNA in the human genome. Nat. Rev. Cancer.

[137] Papapetrou E.P., Schambach A. (2016). Gene insertion into genomic safe harbors for human gene therapy. Mol. Ther..

[138] Fraietta J.A., Nobles C.L., Sammons M.A., Lundh S., Carty S.A., Reich T.J., Cogdill A.P., Morrissette J.J.D., DeNizio J.E., Reddy S. (2018). Disruption of TET2 promotes the therapeutic efficacy of CD19-targeted T cells. Nature.

[139] Ren J., Liu X., Fang C., Jiang S., June C.H., Zhao Y. (2017). Multiplex genome editing to generate universal CAR T cells resistant to PD1 inhibition. Clin. Cancer Res..

[140] Beane J.D., Lee G., Zheng Z., Mendel M., Abate-Daga D., Bharathan M., Black M., Gandhi N., Yu Z., Chandran S. (2015). Clinical scale zinc finger nuclease-mediated gene editing of PD-1 in tumor infiltrating lymphocytes for the treatment of metastatic melanoma. Mol. Ther..

[141] Vong Q., Nye C., Hause R., Clouser C., Jones J., Burleigh S., Borges C.M., Chin M.S.Y., Marco E., Barrera L. (2017). Inhibiting TGFβ signaling in CAR T-cells may significantly enhance efficacy of tumor immunotherapy. Blood.

[142] Menger L., Sledzinska A., Bergerhoff K., Vargas F.A., Smith J., Poirot L., Pule M., Hererro J., Peggs K.S., Quezada S.A. (2016). TALEN-mediated inactivation of PD-1 in tumor-reactive lymphocytes promotes intratumoral T-cell persistence and rejection of established tumors. Cancer Res..

[143] Qasim W., Zhan H., Samarasinghe S., Adams S., Amrolia P., Stafford S., Butler K., Rivat C., Wright G., Somana K. (2017). Molecular remission of infant B-ALL after infusion of universal TALEN gene-edited CAR T cells. Sci. Transl. Med..

[144] Ihry R.J., Worringer K.A., Salick M.R., Frias E., Ho D., Theriault K., Kommineni S., Chen J., Sondey M., Ye C. (2018). p53 inhibits CRISPR-Cas9 engineering in human pluripotent stem cells. Nat. Med..

[145] Haapaniemi E., Botla S., Persson J., Schmierer B., Taipale J. (2018). CRISPR-Cas9 genome editing induces a p53-mediated DNA damage response. Nat. Med..

[146] Ellis J. (2005). Silencing and variegation of gammaretrovirus and lentivirus vectors. Hum. Gene Ther..

[147] Charrier S., Ferrand M., Zerbato M., Precigout G., Viornery A., Bucher-Laurent S., Benkhelifa-Ziyyat S., Merten O.W., Perea J., Galy A. (2011). Quantification of lentiviral vector copy numbers in individual hematopoietic colony-forming cells shows vector dose-dependent effects on the frequency and level of transduction. Gene Ther..

[148] Kustikova O.S., Wahlers A., Kuhlcke K., Stahle B., Zander A.R., Baum C., Fehse B. (2003). Dose finding with retroviral vectors: Correlation of retroviral vector copy numbers in single cells with gene transfer efficiency in a cell population. Blood.

[149] Wahlers A., Schwieger M., Li Z., Meier-Tackmann D., Lindemann C., Eckert H.G., von Laer D., Baum C. (2001). Influence of multiplicity of infection and protein stability on retroviral vector-mediated gene expression in hematopoietic cells. Gene Ther..

[150] Hegde M., Corder A., Chow K.K., Mukherjee M., Ashoori A., Kew Y., Zhang Y.J., Baskin D.S., Merchant F.A., Brawley V.S. (2013). Combinational targeting offsets antigen escape and enhances effector functions of adoptively transferred T cells in glioblastoma. Mol. Ther..

[151] Hegde M., Mukherjee M., Grada Z., Pignata A., Landi D., Navai S.A., Wakefield A., Fousek K., Bielamowicz K., Chow K.K. (2016). Tandem CAR T cells targeting HER2 and IL13Ralpha2 mitigate tumor antigen escape. J. Clin. Investig..

[152] Grada Z., Hegde M., Byrd T., Shaffer D.R., Ghazi A., Brawley V.S., Corder A., Schonfeld K., Koch J., Dotti G. (2013). TanCAR: A novel bispecific chimeric antigen receptor for cancer immunotherapy. Mol. Ther. Nucleic Acids.

[153] Zah E., Lin M.Y., Silva-Benedict A., Jensen M.C., Chen Y.Y. (2016). T cells expressing CD19/CD20 bispecific chimeric antigen receptors prevent antigen escape by malignant B cells. Cancer Immunol. Res..

[154] Yeku O.O., Purdon T.J., Koneru M., Spriggs D., Brentjens R.J. (2017). Armored CAR T cells enhance antitumor efficacy and overcome the tumor microenvironment. Sci. Rep..

[155] Hoyos V., Savoldo B., Quintarelli C., Mahendravada A., Zhang M., Vera J., Heslop H.E., Rooney C.M., Brenner M.K., Dotti G. (2010). Engineering CD19-specific T lymphocytes with interleukin-15 and a suicide gene to enhance their anti-lymphoma/leukemia effects and safety. Leukemia.

[156] Koneru M., Purdon T.J., Spriggs D., Koneru S., Brentjens R.J. (2015). IL-12 secreting tumor-targeted chimeric antigen receptor T cells eradicate ovarian tumors in vivo. Oncoimmunology.

[157] Hu B., Ren J., Luo Y., Keith B., Young R.M., Scholler J., Zhao Y., June C.H. (2017). Augmentation of antitumor immunity by human and mouse CAR T cells secreting IL-18. Cell Rep..

[158] Cherkassky L., Morello A., Villena-Vargas J., Feng Y., Dimitrov D.S., Jones D.R., Sadelain M., Adusumilli P.S. (2016). Human CAR T cells with cell-intrinsic PD-1 checkpoint blockade resist tumor-mediated inhibition. J. Clin. Investig..

[159] Liu X., Ranganathan R., Jiang S., Fang C., Sun J., Kim S., Newick K., Lo A., June C.H., Zhao Y. (2016). A chimeric switch-receptor targeting PD1 augments the efficacy of second-generation CAR T cells in advanced solid tumors. Cancer Res..

[160] Bajgain P., Tawinwung S., D’Elia L., Sukumaran S., Watanabe N., Hoyos V., Lulla P., Brenner M.K., Leen A.M., Vera J.F. (2018). CAR T cell therapy for breast cancer: Harnessing the tumor milieu to drive T cell activation. J. Immunother. Cancer.

[161] Mohammed S., Sukumaran S., Bajgain P., Watanabe N., Heslop H.E., Rooney C.M., Brenner M.K., Fisher W.E., Leen A.M., Vera J.F. (2017). Improving chimeric antigen receptor-modified T cell function by reversing the immunosuppressive tumor microenvironment of pancreatic cancer. Mol. Ther..

[162] Kobold S., Grassmann S., Chaloupka M., Lampert C., Wenk S., Kraus F., Rapp M., Duwell P., Zeng Y., Schmollinger J.C. (2015). Impact of a new fusion receptor on PD-1-mediated immunosuppression in adoptive T cell therapy. J. Natl. Cancer Inst..

[163] Rataj F., Kraus F.B.T., Chaloupka M., Grassmann S., Heise C., Cadilha B.L., Duewell P., Endres S., Kobold S. (2018). PD1-CD28 fusion protein enables CD4^+^ T cell help for adoptive T cell therapy in models of pancreatic cancer and Non-Hodgkin lymphoma. Front. Immunol..

[164] Craddock J.A., Lu A., Bear A., Pule M., Brenner M.K., Rooney C.M., Foster A.E. (2010). Enhanced tumor trafficking of GD2 chimeric antigen receptor T cells by expression of the chemokine receptor CCR2b. J. Immunother..

[165] Moon E.K., Carpenito C., Sun J., Wang L.C., Kapoor V., Predina J., Powell D.J., Riley J.L., June C.H., Albelda S.M. (2011). Expression of a functional CCR2 receptor enhances tumor localization and tumor eradication by retargeted human T cells expressing a mesothelin-specific chimeric antibody receptor. Clin. Cancer Res..

[166] Rapp M., Grassmann S., Chaloupka M., Layritz P., Kruger S., Ormanns S., Rataj F., Janssen K.P., Endres S., Anz D. (2015). C-C chemokine receptor type-4 transduction of T cells enhances interaction with dendritic cells, tumor infiltration and therapeutic efficacy of adoptive T cell transfer. Oncoimmunology.

[167] Caruana I., Savoldo B., Hoyos V., Weber G., Liu H., Kim E.S., Ittmann M.M., Marchetti D., Dotti G. (2015). Heparanase promotes tumor infiltration and antitumor activity of CAR-redirected T lymphocytes. Nat. Med..

[168] Adachi K., Kano Y., Nagai T., Okuyama N., Sakoda Y., Tamada K. (2018). IL-7 and CCL19 expression in CAR-T cells improves immune cell infiltration and CAR-T cell survival in the tumor. Nat. Biotechnol..

[169] Jones B.S., Lamb L.S., Goldman F., Di Stasi A. (2014). Improving the safety of cell therapy products by suicide gene transfer. Front. Pharmacol..

[170] Sun S., Hao H., Yang G., Zhang Y., Fu Y. (2018). Immunotherapy with CAR-modified T cells: Toxicities and overcoming strategies. J. Immunol. Res..

[171] Lanitis E., Poussin M., Klattenhoff A.W., Song D., Sandaltzopoulos R., June C.H., Powell D.J. (2013). Chimeric antigen receptor T Cells with dissociated signaling domains exhibit focused antitumor activity with reduced potential for toxicity in vivo. Cancer Immunol. Res..

[172] Kloss C.C., Condomines M., Cartellieri M., Bachmann M., Sadelain M. (2013). Combinatorial antigen recognition with balanced signaling promotes selective tumor eradication by engineered T cells. Nat. Biotechnol..

[173] Wu C.Y., Roybal K.T., Puchner E.M., Onuffer J., Lim W.A. (2015). Remote control of therapeutic T cells through a small molecule-gated chimeric receptor. Science.

[174] Fedorov V.D., Themeli M., Sadelain M. (2013). PD-1- and CTLA-4-based inhibitory chimeric antigen receptors (iCARs) divert off-target immunotherapy responses. Sci. Transl. Med..

[175] Roybal K.T., Rupp L.J., Morsut L., Walker W.J., McNally K.A., Park J.S., Lim W.A. (2016). Precision tumor recognition by T cells with combinatorial antigen-sensing circuits. Cell.

[176] Roybal K.T., Williams J.Z., Morsut L., Rupp L.J., Kolinko I., Choe J.H., Walker W.J., McNally K.A., Lim W.A. (2016). Engineering T cells with customized therapeutic response programs using Synthetic Notch receptors. Cell.

[177] Cho J.H., Collins J.J., Wong W.W. (2018). Universal chimeric antigen receptors for multiplexed and logical control of T cell responses. Cell.

